# Exploring the influence of electoral political orientation on health outcomes: a scoping review

**DOI:** 10.1590/0102-311XEN051525

**Published:** 2026-04-27

**Authors:** Juan Felipe Rincón Mejía, Antonio Fernando Boing, Marco A. Peres

**Affiliations:** 1 Universidade Federal de Santa Catarina, Florianópolis, Brasil.; 2 National Dental Research Institute, National Dental Centre, Singapore.; 3 Health Services and Population Health Research Programme, Duke-NUS Medical School, Singapore.

**Keywords:** Scoping Review, Voting, Health Status Indicators, Revisão de Escopo, Votação, Indicadores de Saúde, Revisión de Alcance, Votación, Indicadores de Salud

## Abstract

Political orientation has been linked to short-term health outcomes, yet its long-term effects remain underexplored. Given the influence of political contexts on health, understanding these associations is crucial for public health. This scoping review synthesizes the literature on how political orientation (reflected in voting patterns) relates to health indicators, vaccination behavior, and mental health outcomes. Following the Joanna Briggs Institute scoping review methodology and PRISMA-ScR reporting guidelines, a comprehensive search was conducted on PubMed, Embase, Scopus, Web of Science, LILACS, and ProQuest Dissertations and Theses Global, including peer-reviewed and grey literature. A total of 53 studies met the chosen eligibility criteria. The findings show that political orientation significantly shapes health outcomes: more conservative regions show poorer general health indicators, lower vaccination rates, and higher vaccine hesitancy. Political orientation was also associated with increased anxiety, depression, and stress. The included studies employed diverse methodologies (some of which included causal inference techniques to strengthen their analysis). Overall, the results underscore the role of political context in shaping health behaviors and outcomes, emphasizing the need for public health strategies that consider these dynamics.

## Abstract (pt)

A orientação política tem sido ligada a resultados de saúde de curto prazo, mas seus efeitos a longo prazo continuam pouco explorados. Dada a influência dos contextos políticos na saúde, compreender essas associações é crucial para a saúde pública. Esta revisão de escopo sintetiza a literatura sobre como a orientação política (refletida nos padrões de votação) se relaciona com indicadores de saúde, comportamentos de vacinação e resultados de saúde mental. Seguindo a metodologia de revisão de escopo do Instituto Joanna Briggs e as diretrizes de relatório PRISMA-ScR, uma busca abrangente foi realizada no PubMed, Embase, Scopus, Web of Science, LILACS e ProQuest Dissertations and Theses Global, incluindo literatura revisada por pares e literatura cinzenta. No total, 53 estudos atenderam aos critérios de elegibilidade. Os resultados revelam que a orientação política influencia significativamente os resultados de saúde, com regiões mais conservadoras apresentando indicadores gerais de saúde mais baixos, taxas de vacinação mais baixas e maior hesitação vacinal. A orientação política também esteve associada ao aumento da ansiedade, depressão e estresse. Os estudos incluídos empregaram metodologias diversas, algumas das quais usaram técnicas de inferência causal para fortalecer suas análises. No geral, os resultados ressaltam o papel do contexto político na formação dos comportamentos e resultados de saúde, enfatizando a necessidade de estratégias de saúde pública que considerem essas dinâmicas.

## Abstract (es)

La orientación política se ha relacionado con resultados de salud a corto plazo, pero sus efectos a largo plazo siguen sin estar suficientemente estudiados. Dada la influencia de los contextos políticos en la salud, comprender estas asociaciones es fundamental para la salud pública. Esta revisión exploratoria sintetiza la bibliografía existente sobre cómo la orientación política, reflejada en los patrones de voto, se relaciona con los indicadores de salud, los comportamientos de vacunación y los resultados de salud mental. Siguiendo la metodología de revisión exploratoria del Instituto Joanna Briggs y las directrices de presentación de informes PRISMA-ScR, se llevó a cabo una búsqueda exhaustiva en PubMed, Embase, Scopus, Web of Science, LILACS y ProQuest Dissertations and Theses Global, incluyendo la literatura revisada por pares y la literatura gris. Un total de 53 estudios cumplieron los criterios de elegibilidad. Los resultados revelan que la orientación política influye significativamente en los resultados de salud, ya que las regiones más conservadoras presentan indicadores de salud general más deficientes, tasas de vacunación más bajas y una mayor resistencia a las vacunas. Además, la orientación política se asoció con un aumento de la ansiedad, la depresión y el estrés. Los estudios incluidos emplearon diversas metodologías, y algunos de ellos utilizaron técnicas de inferencia causal para reforzar el análisis. En general, los resultados subrayan el papel del contexto político en la configuración de los comportamientos y resultados de salud, y enfatizan la necesidad de estrategias de salud pública que tengan en cuenta estas dinámicas.

## Introduction

Voting patterns serve as a significant indicator of political orientation, reflecting populations’ ideological leanings and policy preferences. While individual political beliefs can be complex, analyzing collective voting behavior concretely measures how communities position themselves along the political spectrum ^1^. Research has indicated that voting behavior is influenced by a combination of collective cognitive, emotional, and social factors. This reflects how stereotypes, values, and group predilections can be activated within the public sphere. As a result, voting behavior serves as a significant indicator for studying societal trends ^2^. This approach enables analyzing how political orientation relates to social and health outcomes. Using voting patterns as a proxy for political orientation is particularly valuable in longitudinal studies as it helps to track changes over time and across different political contexts ^3^.

The relationship between politics and health has gained global attention as societies increasingly recognize how political decisions affect public well-being. Since the early 2000s, interest in the influence of political leadership on health has grown worldwide as political choices shape healthcare policies, medical access, and population health outcomes ^4^. Political orientation significantly influences public health practices across political and economic contexts. In Europe, for example, austerity measures following the 2008 financial crisis had severe health consequences. Studies reveal that policies in countries such as Greece, Spain, and Portugal increased mortality rates and reduced healthcare access, with budget cuts disproportionately harming vulnerable populations and exacerbating health inequalities ^5^ ^,^ ^6^.

The connection between politics and health outcomes is evident globally. In the United Kingdom, austerity policies under conservative governments have been associated with increased mental health problems, rising suicide rates, and higher infant mortality ^7^. In contrast, countries with social democratic traditions, such as Sweden, consistently show better health outcomes due to their emphasis on social welfare and equitable healthcare access ^8^. Similarly, Japan and South Korea illustrate how stable governance and proactive public health policies contribute positively to population health. The universal healthcare and preventive policies in Japan have enhanced life expectancy and reduced infant mortality ^9^, whereas the effective response to COVID-19 in South Korea - via widespread testing, contact tracing, and transparent communication - resulted in comparatively low infection and mortality rates ^10^.

During Donald Trump’s first presidency, the United States faced major shifts in healthcare policy. A key attempt to repeal the *Affordable Care Act* (ACA) underwent a narrow defeat in the Federal Senate, preventing Republicans from enacting broader changes to the healthcare system. The ACA, which expanded Medicaid and prohibited coverage denial for preexisting conditions, significantly improved access to medical care and helped reduce health disparities ^11^.

The reelection of President Donald Trump has brought significant changes to U.S. health policy, including the appointment of Robert F. Kennedy Jr., a known vaccine skeptic, as Secretary of Health and Human Services. His history of promoting vaccine misinformation has raised concerns among public health experts, who fear it could erode trust in immunization programs. As part of his “Make America Healthy Again” initiative, Kennedy Jr. has prioritized chronic disease prevention by alternative nutritional guidelines. However, his endorsement of vitamins over vaccines during a recent measles outbreak has drawn criticism from healthcare professionals, who stress that such measures are unable to replace immunization ^12^.

In Brazil, the relationship between politics and public health gained significant attention after the 2018 presidential election, which brought Jair Messias Bolsonaro, a right-wing and far-right-aligned candidate, to power. Brazil’s vast geographic and demographic diversity has experienced notable shifts in health policy under different political administrations. Bolsonaro’s administration faced strong criticism for its handling of the COVID-19 pandemic, including downplaying the virus and spreading misinformation. These actions contributed to higher mortality rates and increased vaccine hesitancy, showing the risks of political mismanagement in public health ^13^.

Political orientation extends beyond public policy, shaping key social determinants of health such as healthcare access, education, employment, and housing. Research has also highlighted its role in vaccination behaviors and mental health. During the COVID-19 pandemic, countries led by populist leaders who downplayed the virus saw lower vaccination rates and greater vaccine hesitancy ^14^ ^,^ ^15^. Brazil evinced such pattern under President Bolsonaro, in which political rhetoric significantly shaped public attitudes toward vaccination and actions during the pandemic ^13^ ^,^ ^16^.

Voting behavior serves as an important indicator of collective political orientation. However, it should be recognized as an outcome influenced by individual preferences and larger dynamics such as information flows, digital mobilization, and disinformation strategies ^17^ ^,^ ^18^. This perspective emphasizes that voting patterns stem from complex processes shaped by technological, media, and institutional contexts.

Selecting appropriate indicators is essential when examining the relationship between political orientation and health. Traditional measures such as mortality rates, chronic disease incidence, and life expectancy provide valuable data on a population’s overall health and the effects of healthcare policies on prevention and treatment. However, these indicators often take years to reflect changes, sometimes exceeding the study period ^19^. Mental health is equally important, with indicators such as suicide rates and diagnosed mental disorders offering critical views into the impact of social and healthcare policies. For instance, studies have noted that conservative political environments are often associated with higher stigma and reduced access to mental health services, exacerbating mental health issues ^20^.

Vaccination coverage is a key indicator of the effectiveness of preventive healthcare policies, reflecting community commitment to immunization and its capacity to control infectious diseases. Studies have linked political orientation to vaccination behaviors, with populist-leaning regions often showing lower vaccination rates and greater hesitancy ^15^. During COVID-19 pandemic, coverage became even more critical as it reflected awareness of herd immunity and a population’s resilience against public health threats. The stance of political leaders on vaccination played a significant role in shaping public perceptions and behaviors, stressing the importance of understanding these dynamics ^21^.

The relationship between political orientation and health outcomes is complex and extends beyond governmental actions to broader societal trends (expressed by voting patterns). This review explored how voting behaviors - representing collective political and social attitudes - impact health indicators, even when the preferred political parties or candidates are not in power. By focusing on local and regional contexts, this study investigated the correlation between these voting patterns and various health outcomes and their influence on social determinants of health. This approach shifts attention from individual political leaders to the broader community dynamics reflected in electoral behaviors, aligning itself with prior research that highlights the importance of political engagement in shaping public health independently of national policy direction ^22^.

This study sought to fill gaps in the scientific literature by analyzing research on the complex relationship between political orientation, health indicators, and electoral behavior. It aimed to summarize key findings, outline research characteristics, and broadly overview the current knowledge in the field. By doing so, this study contributes to evidence-based policy development and future research independent of specific national or regional contexts.

## Methods

### Eligibility criteria and search strategy

Global research on the relationship between political orientation, voting behavior, and health indicators was examined in this study to understand how political preferences influence health outcomes. Studies from multiple countries on local and national political contexts were included in this review to provide a comprehensive view of these complex interactions.

**Figure 1:**
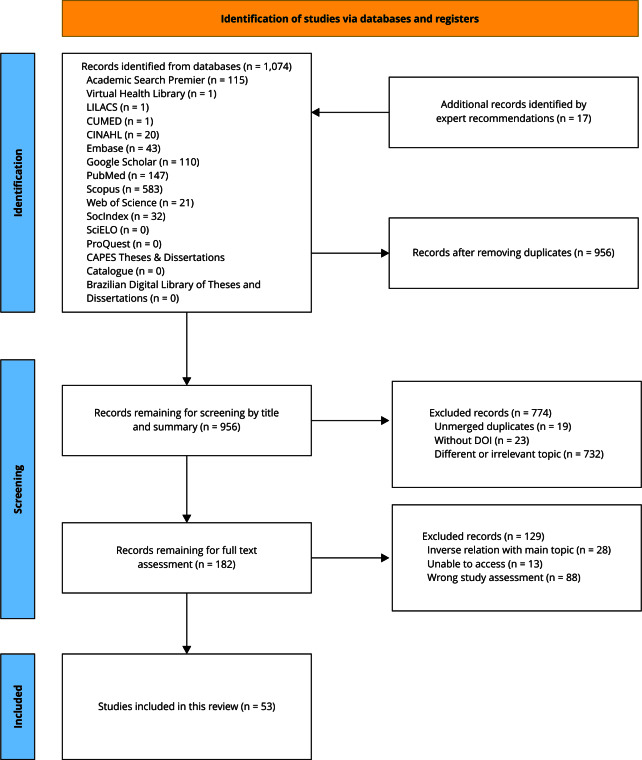
PRISMA flow diagram.

A review protocol was prepared for this study. It is available on the Open Science Framework (https://osf.io/). The protocol can be accessed using the following identifier: https://www.doi.org/10.17605/OSF.IO/K8U4A. The protocol followed the Joanna Briggs Institute methodology ^23^ for scoping reviews and the *PRISMA Extension for Scoping Reviews* (PRISMA-ScR) ^24^. We searched the following bibliographic databases: LILACS, Embase (Elsevier), PubMed/MEDLINE, CINAHL (EBSCO), Web of Science (Clarivate Analytics), Scopus (Elsevier), Google Scholar and SciELO. To capture grey literature, we also searched ProQuest Dissertations, CAPES Theses & Dissertations Catalogue, Theses Global (PQDT Global) and the Brazilian Digital Library of Theses and Dissertations (BDTD). All sources listed in Figure 1 were searched, including those yielding no records. To ensure comprehensiveness, the reference lists of each study included in this review were meticulously examined for any additional relevant research. The search keywords were categorized into “electoral behavior” and “health indicators” to maintain the focus of this study and effectively address the research question in the scoping review (Box 1). The complete search strategies for all databases can be found in the Supplementary Material (Appendix 1; https://cadernos.ensp.fiocruz.br/static//arquivo/suppl-e00051525_4228.pdf).

DOI: digital object identifier.

**Box 1:** Used search terms.

#	TERMS REGARDING CATEGORY
S1	((“electoral behavior” OR “electoral pattern” OR “electoral patterns” OR “Voting behavior” OR “Election influence” OR “Political trust” OR “Election choices” OR “Voter preferences” OR “Political participation” OR “Voting patterns” OR “Election decisions” OR “Political orientation” OR “Political identity” OR “Political beliefs” OR “Ideological affiliation” OR “Political ideology” OR “political views” OR “Political affiliation” OR “Party preferences” OR “Left-wing” OR “Right-wing” OR “Political spectrum” OR “Political values”) AND (“Quality Indicators, Health Care” OR “Health Care Quality Indicators” OR “Health Status Indicators” OR “Health Indicators” OR “Health Indicator” OR “Health index” OR “Health Status Index” OR “Health Status Indexes” OR “Health Status Indices” OR “Health Metric” OR “Health Metrics”))

### Study selection

This scoping review aimed to identify studies on the relationship between political orientation or voting behavior and health outcomes, including physical and mental health and vaccination rates. To account for recent political developments, this review included studies that were published from 2000 to 2025 without language restrictions, ensuring broad geographic coverage. Studies were excluded if they failed to directly address the political orientation-health relationship, lacked theoretical or empirical relevance, or used non-representative and biased samples. Primary research, systematic reviews, and grey literature were included from various sources, such as scientific journals, books, government reports, and academic theses, covering quantitative, qualitative, and theoretical studies. An initial pilot search (2013-2023) proved insufficient, prompting the research team to expand the timeframe to 2000-2025 for a more comprehensive review.

### Summary and report of the results

After completing the literature search, all citations were compiled and deduplicated using Zotero, version 6.0.30 (https://www.zotero.org/), prioritizing entries with comprehensive information and digital object identifiers (DOIs). This resulted in 1,074 unique references and 17 additional expert-recommended articles. In total, 135 duplicates or incomplete records were removed, leaving 956 articles for screening by title and abstract. During screening, 774 articles were excluded: 19 duplicates, 23 without a DOI, and 732 that were irrelevant to the topic of this research. Thus, 182 articles were selected for full-text review. In this stage, 129 articles were further excluded for reasons that included focus on how health indicators influenced voting behaviors (n = 28), lack of open access (n = 13), or differing significantly from the objectives of this review (n = 88). The selection process was carefully monitored to ensure its quality. Ultimately, 53 studies met all inclusion criteria, directly addressing the research objectives and underlining key gaps in the literature. Figure 1 illustrates a PRISMA-ScR flow diagram ^25^ depicting the search process in this study.

### Data charting

The selected articles are summarized in Box 2, which outlines the purpose, target groups, and contexts of each study. Additional details, including study title, keywords, journal, statistical approach, and identified gaps, are provided in the Supplementary Material (Box S1; https://cadernos.ensp.fiocruz.br/static//arquivo/suppl-e00051525_4228.pdf).

**Box 2:** Summary of the selected articles for this study.

STUDY (YEAR)	PURPOSE	GEOGRAPHIC LOCATION	POPULATION EVALUATED	YEAR OF STUDY	METHOD	MAIN FINDINGS
Trimmer ^41^ (2013)	To analyze the relationship between voters, partisan ideology, income, and their impact on national health morbidity and mortality measures	United States	Residents at the county level	2008 and 2012 presidential elections	University of Wisconsin Population Health Institute’s *County Health Rankings* data for analysis	• Significant interactions between income quartiles and partisan categories on health indicators
Lucas ^56^ (2019)	To analyze the impact of the political ideology of mayors on health indicators and health expenditure in municipalities	Brazil	Municipalities in Brazil	Data from 2005 to 2016	Regression discontinuity design	• Significant partisan effect only on FHP coverage • Left-wing municipalities have higher coverage • No significant partisan effect on other health indicators
Rinaldi & Bekker ^46^ (2021)	To map the available empirical evidence regarding the influence of radical right populist parties on welfare policy reforms and to understand how this relationship is mediated by political system in different countries	Europe	Vulnerable population groups; European populations affected by welfare policies	Literature published from 2000 to 2019	Scoping review	• Radical right populist parties’ chauvinistic positions on welfare negatively affect the health of vulnerable population groups • Differences in the implementation of chauvinistic welfare policies mediated by the constitutional order • Welfare chauvinism is more pronounced in countries with tax-based healthcare systems
Subramanian & Perkins ^47^ (2010)	To investigate whether fundamental differences in health status and behaviors exist between individuals who identify with conservative and liberal political parties in the United States	United States	Individuals participating in the *General Social Surveys*	Data from 1972-2006	Weighted binary logistic regression model procedures	• Republicans report lower rates of poor health and are less likely to be smokers than Democrats • These differences persist even after adjusting for several covariates
Bollyky et al. ^69^ (2023)	To identify factors associated with cross-state variation in COVID-19 infection and mortality rates in the U.S. and assess the trade-offs between health outcomes and economic and educational impacts	United States	Population of U.S. states	From January 1, 2020 to July 31, 2022	Regression analysis, data standardization	• Social, economic, and racial inequities were associated with higher COVID-19 infection rates • Political affiliation of state governors failed to significantly affect outcomes • States with higher proportions of Republican voters had worse outcomes
Goodwin et al. ^50^ (2018)	To explore the overlap between the geographic distribution of high opioid use in U.S. counties and votes for the Republican candidate in its 2016 presidential election and to investigate how individual and county-level demographic and economic measures explain this association	United States	Medicare Part D enrollees	Data from 2015 (opioid use) and 2016 (voting data)	Multilevel analysis	• Significant correlation (0.42, p < 0.001) between Republican presidential votes in a county and its adjusted rate of prolonged opioid prescriptions • Support for the Republican candidate constitutes a physical, economic marker associated with opioid use
Krok-Schoen et al. ^64^ (2018)	To examine how demographic, general health, religious, and political influenced beliefs about mandatory school vaccinations and the history of vaccination refusal for children in Ohio Appalachian parents	Ohio Appalachia, United States	Parents (n = 337) of girls aged 9-17 years from 12 counties in rural Ohio Appalachia	2013 and 2014	Multivariate logistic regression models	• 47% of parents believed they should have the right to refuse mandatory school vaccinations • Political affiliation significantly influenced these beliefs. Republicans and Independents were more likely to support the right to refuse than Democrats • 39% of parents reported having refused a vaccine for their child, with significant predictors being the female gender and the belief in the right to refuse school vaccinations
Kim et al. ^30^ (2015)	To examine the effect of civic participation on self-rated health status using a multi-level analysis of data from the *World Value Survey* across 44 countries	44 countries included in the *World Value Survey*	Individuals (n = 50,859) from 44 countries who participated in the survey	2005-2008	Multilevel logistic regression analysis	• People who participated in voting and voluntary social activities reported better self-rated health • Unconventional political participation was negatively associated with subjective health but became insignificant in OECD countries • The democratic index and public health expenditure were significant factors in determining self-rated health • Social participation had a contextual association with subjective health status, with variations between the full sample and OECD countries
Carneiro et al. ^49^ (2022)	To describe the policy orientation of party coalitions, budget outlay, and the structure and performance of medical and dental care	Brazil	Municipalities in Brazil	Data from 2008 to 2013	Regression analysis	• Political coalitions and financial investments significantly influence the performance of primary healthcare services • Municipalities governed by coalitions showed better healthcare outcomes
Bernet ^70^ (2022)	To investigate the association of racial and ethnic composition, segregation, and 2020 presidential election voting results with COVID-19 infections and deaths in Florida counties	Florida, United States	Population of Florida counties	Data collected in March 2021	Poisson regression analysis	• Higher proportions of Black residents experience disproportionately higher COVID-19 infection and mortality rates • Disparities are further inflated in counties with larger Republican vote shares • Hispanic population proportions and segregation are also associated with higher COVID-19 infection and mortality rates in more Republican-leaning counties
Cunningham & Nite ^65^ (2021)	To examine the county-level association between health determinants, demographics, and voting patterns on mask use in the United States during the COVID-19 pandemic	United States (county-level analysis)	County residents across the United States (n = 3,142 counties)	Data collected in July 2020	Two-level random effects regression models	• Health behaviors were positively associated with mask use • The physical environment was negatively associated with mask use • Clinical care and social and economic factors showed no significantly association with mask use • Counties with higher percentages of Democratic voters had higher rates of mask use
Bor ^51^ (2017)	To assess whether voting patterns in the 2016 U.S. presidential election were correlated with long-term trends in county life expectancy	United States	County-level data across the United States	Data from 1980 to 2014 for life expectancy and voting data from the 2008 and 2016 elections	Multivariable regression analysis that was adjusted for county demographic and economic characteristics	• Less support for Trump from counties experiencing greater gains in life
Hrivnák et al. ^42^ (2023)	To estimate the impact of civic political and community engagement on relational and mental health in a medium-sized urban settlement in Slovakia	Nitra, Slovakia	Residents of Nitra, Slovakia (n = 318)	Data collected in 2022	Electronic survey and cross-sectional regression models	• Positive effects of community engagement on relational health • Political participation contributed to reducing depressive symptoms. However, community engagement and mental health showed no significant relationships • Great political engagement negatively impacted relational health
Lee ^26^ (2023)	To empirically analyze the relationship between democracy and public health using robust econometric methods on panel data from 188 countries	Global (n = 188 countries)	Country-level data from 188 countries	Data collected from 1972 to 2019	Pooled ordinary least squares, fixed effects, dynamic generalized method of moments, split-sample methods, and quadratic models	• Democracy positively impacts public health, especially in reducing infant mortality and increasing life expectancy • In a threshold effect, the positive impact of democracy on health is less evident in low-income countries • A U-shaped relationship exists between democracy and infant mortality
Delis et al. ^66^ (2023)	To introduce new indices to measure the efficiency of government policies in dealing with the COVID-19 pandemic and to find the determinants of government policy efficiency	Global (n = 81 countries)	Country-level data from 81 countries	Data from May 2020 to November 2021	Data envelopment analysis and stochastic frontier approach were used to create government policy efficiency indices	• Positive correlation between government policy efficiency and factors such as institutional quality, democratic principles, political stability, and high public health spending • Negative correlation between economic inequality and government policy efficiency
Mukhopadhyay ^60^ (2022)	To examine the effect of the 2020 presidential election on anxiety and depression among Americans using data from the *2020 Household Pulse Survey*	United States	*2020 Household Pulse Survey* (n = 2.45 million)	Data from April 2020 to December 2021	Logistic regressions	• Moderate to severe anxiety and depression increased steadily up to the presidential election • Those symptoms decreased afterward
Arruda & Rocha ^54^ (2024)	To examine the effects of municipal political transitions on the provision of health services and infant health indicators, particularly focusing on critical periods for human development	Brazil	Municipalities in Brazil	Recent Brazilian municipal elections	Causal inference methods	• Political transitions temporarily but significantly reduce health service provision • Infants exposed to these transitions have higher chances of being born with low birth weight • Infants exposed to these transitions have increased infant mortality rates
Beckfield & Krieger ^43^ (2009)	To review and synthesize empirical studies linking political systems and priorities to health inequities and propose a research agenda to address identified gaps	Global, with a focus on the Global North	Various populations across countries	Reviewed studies from 1992 to 2008	Systematic search and review of studies on the ISI Web of Knowledge and PubMed databases	• Transition to capitalism and neoliberal restructuring likely increase health inequities • Welfare states have mixed effects on health inequities • Political incorporation of marginalized groups tends to reduce health inequities
Chioro et al. ^58^ (2023)	To analyze the transition from the Bolsonaro government to the Lula government in Brazil and its implications for the Brazilian health system, focusing on the challenges and opportunities to rebuild and strengthen universal healthcare	Brazil	SUS users	Government transition period from 2022 to 2023	Case study	• Bolsonaro’s administration policies significantly defunded and weakened SUS • Lula’s administration can rebuild SUS, emphasizing the importance of universal health coverage
Duggan ^59^ (2022)	To examine the influence of gender and political orientation on prosocial behavior	Ireland	Irish adults (n = 313)	2022	Hierarchical regression analyses	• Left-wing individuals show higher prosocial behavior than right-wing ones • Women show higher emotional prosocial behavior than men
Matsubayashi & Ueda ^32^ (2012)	To examine the effect of government partisanship on citizens’ well-being, measured by life satisfaction and suicide rates	21 OECD countries	Citizens of 21 OECD countries	1980-2004	Panel data analysis	• Left-leaning and Christian democratic governments are associated with higher life satisfaction and lower suicide rates
Siddiqi et al. ^27^ (2019)	To investigate the rising mortality rates among white individuals in the United States, particularly focusing on the concept of “deaths of despair”, linked to perceived social status threats rather than traditional economic factors	United States	Non-Hispanic White individuals aged 25-54 years	2000-2016	County-level fixed effects model	• Rising mortality in white individuals goes beyond the least educated • Perceptions of social status threats are linked to the increasing Republican voting share
Coleman & Andersson ^33^ (2024)	To investigate how political background (party, ideology, voting policy beliefs) predicts differences in health and well-being indicators during the COVID-19 pandemic	United States	A random sample of 11,000 U.S. households	Data collected in early 2021	Linear regression models	• Republicans report better self-rated health and less distress than Democrats • Liberal beliefs configure robust predictors of health differences • COVID-19 exposures and attitudes significantly mediate these political health differences
Burgess et al. ^44^ (2019)	To characterize the political ideology of first-year medical students and to assess how their political ideology predicts attitudes and beliefs related to the care of marginalized patients by their fourth year of study	United States	Medical students from a stratified random sample with 49 medical schools	2010-2014 follow-up	Mixed-effects linear regression analysis of survey data	• Conservative political ideology in first-year students predicted higher levels of bias and negative attitudes toward marginalized groups in their fourth year of study • Conservative students showed lower motivation to control prejudice and lower empathy
Kelleher et al. ^39^ (2002)	To determine the relationship between mortality patterns, indicators of deprivation, general lifestyle, and social attitudes, as exemplified by general election voting patterns	Ireland	Adults aged over 18 years (n = 273 districts)	Data up to 1997	Correlation and regression analyses	• Relations between left-wing voting and health dissatisfaction and smoking rates • No significant relation between standardized mortality ratios and voting patterns for Fianna Fail and Fine Gael • Fianna Fail voting pattern is inversely related to dissatisfaction with health
Perri ^55^ (2019)	To study the potential effects of implementing Matteo Salvini’s economic program and political strategies on the Italian economy and society	Italy	Italian electorate and society	Data and political events up to 2019	Analysis of economic data, political strategies, communication methods, and electoral trends	• Implementation of Salvinomics could have increased inequalities and economic instability • Institutional reforms could have destabilized Salvini’s electoral base
Curtis et al. ^52^ (2021)	To assess the association between changes in life expectancy and voting patterns in the 2020 U.S. presidential election	United States	Residents of 3110 U.S. counties	Data from 1980 to 2014 for life expectancy and from 2020 for voting patterns	Analysis of county-level life expectancy data and voting results	• Counties with less positive changes in life expectancy were more likely to vote for the Republican candidate • An increase in life expectancy was associated with an increase in the Republican vote share from 2016 to 2020
Coêlho et al. ^57^ (2016)	To explore the diffusion of social health policies in Brazil, specifically its FHP, by the roles of internal and external factors in the adoption of the program by local governments	Brazil	5,560 municipalities in Brazil	Data from 1997 to 2010	Event history analysis	• Political competition and ideology significantly influence FHP adoption • Municipalities with left-wing mayors are more likely to adopt the FHP
Shaw et al. ^61^ (2002)	To investigate the relationship between political regimes and suicide rates, specifically examining how suicide rates have risen during periods of conservative government in Australia and the United Kingdom	Australia and the United Kingdom	Nationwide populations of Australia and the United Kingdom	Data from 1901 to 2000	Comparative analysis of suicide rates	• Higher suicide rates were observed during periods of conservative government in Australia • The effect was strongest when both levels of government were conservative • Similar patterns were observed in the United Kingdom
Castilho et al. ^71^ (2023)	To study the role of political orientation (particularly support for President Bolsonaro) in the mortality rates of COVID-19 in Brazil	Brazil	Municipalities in Brazil	Data from COVID-19 pandemic in 2020 to October 2021	Econometric models	• Higher COVID-19 mortality rates in municipalities with higher support for Bolsonaro • Population mobility is a significant transmission channel for the disease • Political denialism fails to significantly affect complete vaccination rates
Gollwitzer et al. ^62^ (2020)	To investigate how political partisanship affects physical distancing behaviors during the COVID-19 pandemic and its subsequent impact on infection and fatality growth rates	United States	Approximately 3,025 U.S. counties	Data from March to May 2020	Multilevel mixed-effects models, mediation analyses, geotracking data	• Counties that voted for Trump showed 14% less physical distancing than those that voted for Clinton • Partisanship was more strongly associated with physical distancing than other factors such as population density and median income • Reduced physical distancing in pro-Trump counties was linked to higher infection and fatality growth rates
Muntaner et al. ^36^ (2011)	To determine the impact of political and welfare state variables on infant and child health outcomes	Wealthy OECD countries (n = 19)	Populations of 19 wealthy OECD countries	1960-1994	Time-series multivariate regression model	• Political and welfare variables are associated with infant and child health indicators • Votes obtained by social democratic or labor parties are related with low birth weight rates
Subramanian et al. ^31^ (2010)	To examine the association between political ideology and health status in Japan, investigating whether individuals with conservative political beliefs report better health and lower smoking rates than those with progressive beliefs	Japan	Individuals from the *Japan General Social Survey* from 2000 to 2006	Data from the 2000-2003, 2005, and 2006 *Japan General Social Surveys*	Logistic regression models	• Inverse association between political ideology and self-rated poor health and smoking status • Conservatives were less likely to report poor health (OR = 0.86) and less likely to smoke (OR = 0.80) • Political ideology might configure a marker for several latent values and attitudes that benefit health
Barbieri & Bonini ^63^ (2021)	To document how political orientation influences adherence to social distancing measures during the COVID-19 pandemic in Italy using province-level geolocation data	Italy	Residents in Italian provinces	Data from February to June 2020	Econometric modeling	• Provinces with higher support for extreme right-wing parties showed lower compliance with social distancing measures • Provinces with higher protest votes similarly showed lower compliance with social distancing • Higher compliance with social distancing was observed in provinces with more support for Five Star Movement • Political (mis)belief and discontent significantly influenced compliance with government lockdown measures
Kannan & Veazie ^35^ (2018)	To examine the associations of political orientation and political with various health behaviors in the United States	United States	Adult sample from the *Annenberg National Health Communication Survey*	Data from 2005 to 2012	Logistic regression models	• Democrats/liberals had higher odds of cigarette smoking and excessive drinking than Republicans/conservatives • Republicans/conservatives ate fewer servings and varieties of fruit and vegetables, ate more high-fat and processed foods, and engaged in less in-depth health information searches than Democrats/liberals • Conservatives had lower odds of exercise participation than liberals. Republicans had lower odds of flu vaccination
Hsiehchen et al. ^67^ (2020)	To evaluate the relationship between compliance to non-pharmaceutical interventions and political party affiliations, specifically focusing on Republican identification	United States	Individuals residing in several U.S. states during the COVID-19 pandemic	March and April 2020	Multivariable linear regression models to determine the impact of Republican proportion and voter support for President Trump	• Negative correlation between Republican affiliation and non-pharmaceutical intervention compliance • Highlighted impact of political orientation on mobility restrictions
Tapia Granados ^40^ (2010)	To analyze the influence of political regimes (social democracies vs. right-wing governments) on mortality decline in eight European countries	Eight European countries	General population of eight European countries	From 1950 to 2000	Analysis of mortality rates and life expectancy data from various sources	• Significant convergence in population health indicators in the eight countries despite differing political regimes • Mean decadal gains in longevity were higher in Southern European countries • Political regime, health expenditure, and type of welfare state do not appear to be major determinants of mortality decline
Navarro et al. ^45^ (2006)	To examine the complex interactions between political traditions, policies, and public health outcomes and to determine whether different political traditions have been associated with systematic patterns in population health over time	Wealthy OECD countries	Populations of 19 wealthy OECD countries	1950 to 2000	Heuristic framework and bivariate Pearson correlation coefficients to political, economic, social, and health variables	• Political ideologies of governing parties significantly affect health indicators • Parties with egalitarian ideologies tend to implement redistributive policies, leading to better health outcomes • Redistributive policies are positively associated with health outcomes
Cinaroglu ^34^ (2019)	To determine the relationship between politics, labor, welfare state indicators, economic inequality, and health outcome indicators	Türkiye	Population of the 81 provinces of Türkiye	Data from 2015	Path analytic model	• A significant relationship exists between voter partisanship and health outcomes • Increased voter support for the ruling party (AKP) is associated with higher health services
Alexiou & Trachanas ^37^ (2021)	To explore the relationship between government political party orientation and infant mortality, examining how different political orientations influence health outcomes by government health expenditure	15 countries from the G20 group	Populations of the 15 G20 countries	Data from 2000 to 2018	Panel quantile	• Political party orientation significantly affects health outcomes • Left-wing parties are associated with better health outcomes • Government health expenditure plays a main role in reducing child mortality • Redistributive policies contribute to better health outcomes
Lima et al. ^28^ (2024)	To evaluate the association between excess mortality and political partisanship in Brazil using municipal death certificates and first-round electoral results of presidential elections in 2018 and 2022	Brazil	Brazilian municipalities	Data from 2020 to 2021	Spatial regression models and correlation analysis	• Positive association between the percentage of votes for Bolsonaro and excess mortality in Brazilian municipalities during the COVID-19 pandemic • This association suggests that Bolsonaro’s public stance and rhetoric against pandemic measures influenced the excess mortality rates
Kimball & Wissner ^38^ (2015)	To explore the relationships between social determinants of health (including religion, voting patterns, child poverty, and income inequality) on women’s reproductive health outcomes in the United States	United States	State-level populations across the 50 U.S. states	Data from 2007-2008	Secondary data analysis using state-level data	• Higher infant mortality rates are associated with higher religiosity scores • Lower abortion rates are associated with voting conservatively • Higher teen birth rates are associated with higher child poverty rates and voting conservatively
Mackenbach & McKee ^48^ (2013)	To examine the effects of social-democratic government participation on indicators of preventive health policy and population health outcomes in Europe	Various European countries	General population of the European countries	Data from 1946 to 2008	Regression models	• Social-democratic countries tend to have better health outcomes • Health indicators such as male smoking prevalence, alcohol consumption, and road safety measures were better in social-democratic countries
Morey et al. ^29^ (2021)	To examine the effect of the 2016 U.S. presidential election on the mental health of Latin and White populations considering race/ethnicity, language of interview, and state-level support for Trump or Clinton	United States	Participants from BRFSS survey	Data from 2011 to 2018	Difference-in-differences analysis using negative binomial regression models	• White populations in Clinton states reported more poor mental health days in response to the post-election period than White populations in Trump states • English-speaking Latin people living in Trump states experienced higher than expected poor mental health • Spanish-speaking Latin people, by contrast, reported less poor mental health
Kohler & Koinig ^80^ (2023)	To examine the relationship between science-related populism and individuals’ attitudes toward vaccination, hypothesizing that science-related populism influences individual responses toward different vaccinations	Germany and Austria	Participants from Germany and Austria (n = 870)	2021	Binomial logistic regressions, linear regression models	• Science-related populism significantly influences attitudes toward COVID-19 and measles, mumps, and rubella vaccinations • Decreased vaccination confidence and collective responsibility • Increased complacency and perceived constraints
Sabahelzain et al. ^68^ (2021)	To examine how political factors such as government handling of the pandemic, populism, and vaccine nationalism influence public confidence in COVID-19 vaccines	Philippines, Brazil, the United States, and European nations	General populations in the mentioned countries	2021	Qualitative analysis	• Public confidence in COVID-19 vaccines is significantly influenced by perceptions of government handling of the pandemic
Hamamsy et al. ^53^ (2021)	To comprehensively analyze the relationship between voting patterns and over 150 public health and wellbeing variables in U.S. counties	United States	U.S. counties	Data from 1980 to 2016	Pearson correlations, linear mixed models, multivariate linear models, lasso regression	• Counties voting Republican in the 2016 election had worse overall health outcomes than those voting Democrat • Great significant associations between various health measures and voting patterns
Martín-Martín et al. ^77^ (2024)	To estimate the effects of political turnover on municipal health indicators and test whether changes in party leadership at the municipal level influence public health policies and workforce composition	80 countries on five continents	National populations of the 80 included countries	2024	Multiple linear regression models analyzing excess mortality, democracy indices, and control variables (health spending, overweight population, population over 65, globalization index, income levels, temperature)	• Higher democratic quality is associated with lower excess mortality during the pandemic • Political culture had a strongest inverse correlation with excess mortality (-326.50, p < 0.001) • Countries with lower democracy index values had higher excess mortality • Government function, electoral process, and civil liberties also showed significant inverse relationships with mortality rates
Phifer ^78^ (2024)	To investigate whether political orientation influences sugar consumption	United States	General U.S. consumers	2024	Five empirical studies testing the relationship between political orientation and sugar intake. Three additional studies assessing how liberals and conservatives react to sugar-reducing nudges	• No significant differences in sugar consumption between conservatives and liberals • Political ideology affect reactions to sugar-reducing nudges • Conservatives resist nudges that restrict personal choice (e.g., school bans, taxation) • Liberals support interventions that promote healthier diets more
Oberlander ^17^ (2024)	To examine how polarization and partisanship shape health policy in the U.S and investigate how political ideology influences public health behaviors and healthcare access	United States	General U.S. population	2024	Evaluates how health outcomes differ across partisan lines in several states. Examines political rhetoric, media framing, and legislative debates around healthcare	• Political polarization has reshaped U.S. health policy, creating two-tiered healthcare systems between Republican and Democratic states • Democratic-led states enacted stricter public health mandates (masking, social distancing, vaccine requirements) • Republican-led states opposed federal mandates, emphasizing “personal freedom” over public health measures •Higher vaccination rates among Democrats; lower rates in Republican-majority counties.
De Araújo et al. ^76^ (2024)	To estimate the effects of political turnover on municipal health indicators, and test whether changes in party leadership at the municipal level influence public health policies and workforce composition	Brazil	Brazilian residents	2024	Regression discontinuity design applied to close municipal elections (margin of ±5%). Analysis of health indicators (SUS workforce, immunization, primary care coverage, etc.)	• Political turnover improves administrative health indicators (e.g., workforce numbers, primary care coverage) but not structural health outcomes (e.g., mortality rates) • Municipalities with turnover had better-qualified health managers • Increased establishment of basic health units in municipalities that experienced political turnover
Arena et al. ^79^ (2024)	To examine how population health status correlates with voter turnout in the 2020 U.S. presidential election. It also introduces and validates a LHI as a predictor of civic engagement	United States	All U.S. counties (n = 3,062)	2024	County-level health and voting data analysis from multiple public databases, with statistical correlations between LHI scores and 2020 voter turnout. The American Nations regional culture model to geospatially map voter turnout and health disparities	• Higher rates of chronic disease and unhealthy behaviors were strongly linked to lower voter turnout • Republican-majority counties had higher LHI scores and lower voter turnout • Democratic-majority counties had lower LHI scores and higher voter turnout • The relationship between health and civic participation was consistent across all U.S. regional cultures

BRFSS: Behavioral Risk Factor Surveillance System; FHP: Family Health Program; LHI: Lifestyle Health Index; OECD: Organisation for Economic Co-operation and Development; SUS: Brazilian Unified National Health System.

### Use of AI tools

During the preparation of this work, the authors used OpenAI ChatGPT 4.5 (https://openai.com) to improve the writing process. After using this tool/service, the authors reviewed and edited the content as needed, taking full responsibility for the content of this publication.

## Results

### Studies characteristics

A total of 53 articles were selected and organized into three categories: the relationship between political orientation and general health indicators, vaccination behaviors, and mental health outcomes. This structure explained how political factors influence various public health dimensions. Studies assessed diverse geographic contexts, mainly the United States (n = 16), Europe (n = 10), global multi-country analyses (n = 11), Brazil (n = 8), Japan (n = 1), and Australia (n = 1). Methodological approaches varied, ranging from focused analyses on vaccination or mental health to broader evaluations of general health indicators and policy impacts. Findings consistently showed that political orientation significantly affects health outcomes, with conservative-leaning regions often experiencing poorer indicators. Methodologies included regression analysis (n = 15), econometric models (n = 7), multilevel analyses (n = 5), correlation analysis (n = 4), causal inference methods such as difference-in-differences (n = 4), scoping reviews (n = 2), and mixed or other statistical approaches (n = 5). Recent studies with causal inference have furthered the understanding of how political orientation shapes public health outcomes.

Lee ^26^ analyzed the impact of democracy on public health across 188 countries using fixed effects, dynamic generalized method of moments, and other econometric methods. Their findings indicate that democracy is associated with lower infant mortality and higher life expectancy, although the benefits diminish in low-income countries, suggesting a threshold effect. Similarly, Siddiqi et al. ^27^ applied county-level fixed effects models to investigate rising mortality among White Americans from 2000 to 2016, linking these “deaths of despair” to perceptions of social status decline rather than economic hardship. That study found that counties with increasing Republican vote shares showed higher mortality rates, pointing to broader psychosocial stressors influencing health. In Brazil, Lima et al. ^28^ used spatial regression models to assess excess mortality during the COVID-19 pandemic in municipalities with high support for Bolsonaro. Their results showed a clear association between political alignment and pandemic-related deaths, suggesting that political rhetoric and governance choices significantly influenced mortality outcomes. A related study by Morey et al. ^29^ used difference-in-differences analysis to examine the mental health effects of the 2016 U.S. presidential election. Their findings evinced that White individuals in Bill Clinton-supporting states reported increased mental distress post-election, whereas English-speaking Latinx populations in Trump-supporting states experienced heightened psychological stress, threatening mental health responses. These studies provided compelling evidence that political decision-making and ideological divisions tangibly shape public health.

The chosen research included diverse populations, from general county or municipal communities to specific groups such as rural or vulnerable populations impacted by political policies. Counties and municipalities constituted the primary units of analysis - county-level studies were common in the United States, whereas municipal-level studies predominated in Brazil. This local focus could assess in detail how political orientation and voting behaviors affect health outcomes within specific geographic contexts. Selected articles emerged in prominent peer-reviewed journals, including *The Lancet*, the *International Journal of Epidemiology*, *JAMA Network Open*, and the *American Journal of Public Health*.

### Political orientation and health indicators

This review found that mortality rates, self-rated health, mental health, and vaccination coverage comprised the most frequently analyzed outcomes, emphasizing the significant influence of political orientation on public health. Several studies indicated conservative-leaning regions experienced poorer health outcomes and lower vaccination rates, with notably strong associations in mental health and vaccination behaviors. Unconventional political participation was linked to poorer subjective health, although this association was weaker in Organisation for Economic Co-operation and Development (OECD) countries ^30^. Democracy levels and public health spending also emerged as key determinants of health. Additionally, a Japanese study reported that conservatives were less likely to self-report poor health or smoking ^31^, whereas another study found that left-leaning and Christian democratic governments were correlated with higher life satisfaction and lower suicide rates ^32^.

Coleman & Andersson ^33^ investigated how political affiliation, ideology, and voting behavior affected health and well-being during the COVID-19 pandemic, finding that Republicans reported better self-rated health and lower distress than Democrats. A study in Türkiye ^34^ showed significant links between voter partisanship, employment rates, social security satisfaction, and health outcomes, emphasizing the role of inclusive policies. Another U.S study ^35^ explored health behaviors, noting that Democrats and liberals were more likely to smoke and consume alcohol excessively, whereas Republicans and conservatives ate fewer fruits, and vegetables, consumed more high-fat and processed foods, and engaged less actively with health-related information.

Research emphasizes that committed political leadership advocating equal healthcare access is crucial for improving national health outcomes ^36^. One study ^37^ examined how government political orientation (right, center, left) affects infant mortality via healthcare spending, finding that left-wing parties generally produced better health results. A 2015 study ^38^ noted higher infant mortality rates in regions with greater religiosity, correlating conservative voting with lower abortion rates, higher teen birth rates, and increased child poverty. Votes for social democratic or labor parties were associated with lower rates of low birth weight. In Ireland, left-wing voting correlated with higher health dissatisfaction and increased smoking rates.

No significant relationship emerged between standardized mortality ratios (SMR) and voting patterns for Fianna Fail and Fine Gael ^39^. A study explored how different political systems (social democracies vs. right-wing governments) affected mortality decline in eight European countries, finding that political systems, healthcare expenditures, and welfare structures had no significant impact in it ^40^. In a study in Brazil, researchers examined excess mortality in relation to voting patterns from the 2018 presidential election. Its findings indicated that municipalities with higher support for president Bolsonaro experienced higher-than-expected deaths during the COVID-19 pandemic, suggesting that his rhetoric and stance against pandemic measures may have worsened mortality outcomes ^28^.

A series of 12 studies ^26^ ^,^ ^36^ ^,^ ^41^ ^,^ ^42^ ^,^ ^43^ ^,^ ^44^ ^,^ ^45^ ^,^ ^46^ ^,^ ^47^ ^,^ ^48^ ^,^ ^49^ ^,^ ^50^ explored how political orientation and governance shape health outcomes. Their results consistently indicate that conservative political ideology is associated with poorer health outcomes, higher biases among medical students toward marginalized groups, and greater health inequities under neoliberal policies. Conversely, social-democratic approaches were linked to lower infant mortality, longer life expectancy, and improved health policies. Populations that experienced greater political inclusion showed reduced health disparities, whereas populist and conservative ideologies often intensified inequities. In the U.S., Republicans reported better self-rated health and lower smoking rates but counties with strong Republican support in 2016 had higher opioid usage, highlighting complex interactions between health and political beliefs.

Several studies have explored how voting patterns relate to health outcomes, especially life expectancy and overall well-being. Research in the United States found that counties with greater improvements in life expectancy had lower support for Trump in the 2016 election ^51^. Similarly, counties with smaller gains in life expectancy were more likely to support Republicans in 2020, with increasing life expectancy associated with higher Republican vote shares from 2016 to 2020 ^52^. A comprehensive study ^53^ examining over 150 public health indicators found poorer health outcomes in Republican-voting counties, especially in battleground states and areas shifting from Democrat to Republican. Another study ^54^ showed that local political transitions temporarily disrupted healthcare services, negatively affecting infant health indicators such as birth weight and mortality rates.

Outside the U.S., research on Matteo Salvini’s economic policies (“Salvinomics”) suggested that these policies could increase inequality and economic instability, potentially weakening Salvini’s political support via an institutional reform ^55^. In Brazil, a study showed that left-wing municipalities showed higher coverage under the Family Health Program (FHP), finding no clear partisan effects for other health indicators ^56^.

Research also studied the transition from the Bolsonaro government to the Luiz Inácio Lula da Silva government in Brazil, focusing on the defunding and weakening of the Brazilian Unified National Health System (SUS, acronym in Portuguese) under Bolsonaro. The Lula administration rebuilt the SUS and emphasized the importance of universal health coverage. That study also explored the diffusion of social health policies, specifically the Brazilian FHP, and showed that political competition and ideology significantly influence the adoption of that FHP. Municipalities with left-wing mayors were more likely to adopt the program, showing the impact of political alignment and demographic factors on policy adoption ^57^ ^,^ ^58^.

### Political orientation and mental health indicators

The chosen studies examined various mental health indicators and their connections to political factors. A study analyzed rising mortality rates in White Americans ^27^, focusing on “deaths of despair” linked to perceived threats to social status. Its findings indicated that increasing mortality rates went beyond the least educated and correlated a greater sense of social status threat with higher Republican voting shares. Another study explored the relationship between gender, political beliefs, and prosocial behavior, evincing that left-wing individuals showed higher levels of prosocial behavior than right-wing individuals, whereas women had more emotional prosocial behavior than men ^59^.

A study analyzed the impact of the 2020 U.S. presidential election on anxiety and depression using data from the *2020 Household Pulse Survey* ^60^. It found that moderate to severe anxiety and depression increased steadily leading up to the election and declined afterward. Another study examined the relationship between political regimes and suicide rates in Australia and the United Kingdom ^61^, finding higher suicide rates in conservative governments, with the effect being strongest when both national and local governments were conservative. Another study investigated the mental health effects of the 2016 U.S. presidential election on Latinx and White populations ^29^. It found that white individuals in Clinton-supporting states reported more poor mental health days following the election than those in Trump-supporting states. Additionally, English-speaking Latinx individuals in Trump-supporting states experienced higher-than-expected mental health distress, whereas Spanish-speaking Latinx individuals reported fewer poor mental health days.

### Political orientation and vaccination

Several studies explored the relationship between political affiliation and adherence to physical distancing measures during the COVID-19 pandemic and its impact on infection and fatality rates. A study in the United States found that counties that voted for Trump practiced 14% less physical distancing than those that supported Clinton ^62^. Political affiliation showed a stronger association with distancing behaviors than factors such as population density or median income. Additionally, reduced physical distancing in pro-Trump counties was linked to higher infection and fatality growth rates. A study in Italy analyzed the impact of political orientation on adherence to social distancing measures using province-level geolocation data ^63^. Its findings revealed that provinces with stronger support for extreme right-wing parties and higher protest votes had lower compliance with social distancing measures. In contrast, provinces with greater support for the Five Star Movement showed higher adherence. That study concluded that political beliefs and public discontent played a significant role in shaping compliance with government-imposed lockdown measures.

Several studies examined factors influencing vaccination beliefs, compliance with COVID-19 measures, and the role of political affiliation in public health behaviors. A study on Ohio Appalachian (United States) parents found that 47% believed they should have the right to refuse mandatory school vaccinations, with political affiliation significantly shaping these attitudes ^64^. Another study on mask-wearing behavior in the U.S. during the COVID-19 pandemic showed that health-conscious behaviors were positively associated with mask use, whereas environmental factors had a negative influence on it ^65^. Counties with higher percentages of Democratic voters showed higher mask-wearing rates. New indices assessing the effectiveness of government responses to COVID-19 highlighted a positive relationship between policy efficiency, institutional quality, and public health spending, while economic inequality was negatively correlated with policy success ^66^.

Political affiliation also played a crucial role in adherence to non-pharmaceutical interventions, with Republican affiliation linked to lower compliance. Additionally, science-related populism significantly impacted confidence in COVID-19 and measles, mumps, and rubella (MMR) vaccines, and government handling of the pandemic shaped public trust in vaccination efforts. That study also found that science-related populism significantly impacted people’s confidence in COVID-19 and MMR vaccinations. Additionally, it noted that government management of the pandemic and political factors significantly influenced public confidence in COVID-19 vaccines ^67^ ^,^ ^68^. Social, economic, and racial disparities were also associated with higher COVID-19 infection rates, with states that had larger Republican voting shares experiencing worse outcomes. A study of Florida counties (United States) found that higher proportions of Black and Hispanic residents, combined with larger Republican vote shares, were linked to higher infection and mortality rates ^69^ ^,^ ^70^. In Brazil, an analysis of COVID-19 mortality rates showed that municipalities with greater support for Bolsonaro had higher mortality rates ^71^. The study also noted that population mobility played a key role in disease transmission, whereas political denialism failed to significantly impact complete vaccination rates.

### Impact of health on political orientation

This study focused on how political orientation influences health outcomes (rather than the opposite) to assess the extent to which ideologies, party affiliation, and governance shape health behaviors, policies, and system outcomes. This approach helped this research to avoid conflating causality in bidirectional relationships. While this systematic analysis excluded research on health as a determinant of political behavior, it included some references for context. The broader literature acknowledges that health disparities can influence political behavior, such as voter turnout and policy preferences. However, the primary analysis in this study ignored these studies as it focused on how political orientation shapes health-related outcomes.

For example, the article named *Health as an Independent Predictor of the 2017 French Presidential Voting Behavior: A Cross-Sectional Analysis* explored how individual health conditions influenced voting choices in France ^72^. Similarly, *Deaths of Despair and Brexit Votes: Cross-Local Authority Statistical Analysis in England and Wales* examined how regions with worsening health indicators, such as rising mortality from substance use and economic distress, showed distinct voting patterns during the Brexit referendum ^73^. Likewise, *Swing Voting in the 2016 Presidential Election in Counties Where Midlife Mortality Has Been Rising in White Non-Hispanic Americans* suggested that worsening health conditions may have contributed to shifts in political preferences in the U.S ^74^.

Another study, *Independent Relationship of Changes in Death Rates with Changes in U.S. Presidential Voting* ^75^, established correlations between demographic health trends and shifts in electoral preferences over time. These studies, while important in understanding the broader interplay between health and political engagement, fall outside the scope of this study, which focuses on how political ideology and governance influence health outcomes (rather than the reverse relationship). While acknowledging the relevance of these perspectives, we opted to exclude them to maintain a clear analytical boundary and prevent this study from becoming overly expansive.

## Discussion

This scoping review found 53 studies on the relationship between political orientation, health indicators, electoral behavior, and vaccination. The findings illustrate the significant influence of political ideology on health outcomes and behaviors and describe how health conditions shape civic participation and policy preferences.

### Causal pathways linking political orientation and health outcomes

A key finding in the literature refers to the need for a systematic theoretical framework to explain how political orientation influences public health. Democratic governance, policy decisions, and individual political behaviors interact via multiple pathways to shape health outcomes:

#### Policy determinants of health

Political ideologies significantly influence government priorities regarding healthcare access, resource allocation, and preventive health strategies. Conservative administrations often prioritize market-driven healthcare models, whereas progressive or social-democratic governments typically defend expanded public health coverage and policies that address social determinants of health ^41^ ^,^ ^46^ ^,^ ^56^.

#### Public trust and compliance

Political orientation shapes trust in institutions and adherence to public health recommendations. Studies suggest that populations in line with conservative ideologies show greater skepticism toward government-led health interventions, affecting vaccination rates and responses to pandemic mitigation measures ^30^ ^,^ ^49^ ^,^ ^50^ ^,^ ^51^.

#### Structural and behavioral mediators

Health disparities arise from structural inequalities embedded within political systems. Electoral outcomes can influence health policy priorities and the distribution of public health resources within current healthcare systems, potentially leading to regional disparities in morbidity and mortality over time ^32^ ^,^ ^43^ ^,^ ^58^.

### Democratic governance and health resilience

Strong democratic institutions that prioritize transparency, accountability, and civic engagement can lead to better public health outcomes, especially during crises such as the COVID-19 pandemic. However, this relationship is far from universal. Various political systems, including non-liberal regimes, have also achieved positive health results by centralized governance, effective resource mobilization, and robust public health infrastructure, as in countries such as China, Cuba, Japan, and South Korea ^9^ ^,^ ^10^. This indicates that quality of governance, institutional capacity, and social trust may be just as important as the formal political system in determining health resilience ^27^ ^,^ ^33^ ^,^ ^44^.

Political polarization significantly challenges the implementation of equitable health policies. The growing ideological divide shapes attitudes toward public health interventions, influencing individual behaviors and State decisions. Partisan resistance to measures such as vaccination campaigns and regulations on noncommunicable diseases shows how political identity can mediate health behaviors. This suggests that the success of public health interventions depends on scientific evidence and on how these measures are politically framed ^52^ ^,^ ^55^.

### Electoral cycles and health policy stability

Municipal political turnover can serve as a driver of health policy innovation and a source of instability. Intense electoral competition can motivate political leaders to implement noticeable health policy measures. However, the direction of these changes largely depends on the ideological orientation of the new administration, which may either enhance or limit workforce capacity and service provision. Frequent turnover can disrupt policy continuity, limiting the long-term effectiveness of health interventions. While electoral accountability is crucial for governance, ensuring that health policies remain insulated from short-term political fluctuations is essential for sustainable health outcomes ^31^ ^,^ ^57^ ^,^ ^76^.

### Mental health and political climate

An increasing body of literature suggests that politically charged events and shifts in governance can exacerbate mental health issues, contributing to heightened levels of anxiety, depression, and stress. Findings indicate that political instability can have differential effects across demographic groups, with individuals identifying with opposition parties experiencing greater psychological distress ^59^ ^,^ ^60^ ^,^ ^61^. Understanding these effects is crucial for developing interventions that address the intersection of political stress and mental health outcomes.

### Implications for public health policy and political strategy

The evidence suggests that bridging ideological divides in health policymaking requires reframing health interventions in ways that resonate across political orientations. Public health messaging should prioritize community well-being and collective responsibility, describing health as a communal outcome influenced by social determinants and policy contexts rather than depending only on individual behavior change. Policymakers must also address structural barriers to civic engagement, recognizing that public health and democratic participation are mutually reinforcing.

The relationship between political orientation, health outcomes, and electoral behavior is complex and deeply interconnected. This review shows how democratic governance and political ideology shape health resilience, whereas health disparities influence political engagement. Further research should explore the causal mechanisms behind these dynamics, considering factors such as media influence, socioeconomic status, and policy legacies. Integrating insights from public health, political science, and behavioral psychology can help to develop more effective strategies to reduce health inequities and strengthen democratic participation ^28^ ^,^ ^31^ ^,^ ^34^ ^,^ ^36^ ^,^ ^37^ ^,^ ^38^ ^,^ ^48^ ^,^ ^53^ ^,^ ^66^ ^,^ ^67^ ^,^ ^68^ ^,^ ^71^ ^,^ ^76^ ^,^ ^77^ ^,^ ^78^ ^,^ ^79^ ^,^ ^80^.

Some limitations deserve mention. The methodologies and contexts across the included studies made direct comparisons challenging and limited the ability of this study to draw broad conclusions across their settings. Still, this study has notable strengths. Its high methodological rigor followed a registered protocol to ensure transparency and replicability. The entire process adhered strictly to PRISMA-ScR guidelines, providing a structured framework for finding, selecting, and analyzing the literature.

This review has important practical implications. Public health policies and interventions should consider political contexts to enhance effectiveness. For instance, targeted communication strategies could address vaccine hesitancy in conservative regions, whereas areas experiencing heightened political stress could prioritize mental health support. These findings emphasize the need for politically responsive public health strategies that promote more effective and equitable outcomes. This study contributes to the understanding of how political determinants influence public health, highlighting the importance of examining how political orientation shapes health behaviors and outcomes. Future research should focus on local contexts within different countries, particularly Brazil, to develop more nuanced and effective public health strategies. Expanding the scope to include a wider range of health indicators and political contexts would also provide a more comprehensive perspective on the political determinants of health. Despite its systematic and transparent approach, the outlined limitations in this study may have influenced its findings, requiring considered when applied to policy or further research.

This review found a significant gap in the literature regarding the connection between political beliefs and health outcomes, particularly in mental health and vaccination. The limited studies in the area often required consideration of local contexts, especially in Brazil. The lack of comprehensive, context-specific research hinders a full understanding of how political beliefs shape mental health and vaccination behaviors across different populations.

## Final considerations and conclusions

This scoping review synthesized the literature on the relationship between political orientation, health indicators, electoral behavior, and vaccination coverage. Analyzing 53 selected studies, it shows key trends in how political structures shape public health and acknowledges the complexity and potential bidirectionality of these relationships. As a scoping review, rather than formally assessing methodological quality, it maps evidence to inform future research and policy discussions.

The findings describe strong associations between political orientation and health outcomes, although the mechanisms driving these relationships require further exploration. Research suggests that conservative-leaning regions often experience poorer health outcomes than liberal areas, largely due to differences in public health policies, resource allocation, and healthcare access. However, the potential for reverse causality - in which health status influences political behavior - remains an area for further investigations.

A well-substantiated pattern refers to the link between political beliefs and vaccination behavior. Studies show that conservative-leaning regions evince lower vaccine uptake and greater vaccine hesitancy, particularly during the COVID-19 pandemic, emphasizing the need for public health communication strategies that overcome ideological divides.

Another notable trend refers to the impact of political events on mental health. Periods of political instability and electoral polarization have been associated with increased anxiety, depression, and psychological distress across various demographic groups. These findings suggest that political environments directly and indirectly affect mental well-being, reinforcing the need for integrated policies that address health systems and broader social determinants of health.

## Data Availability

The research data are available upon request to the corresponding author.
