# Supplementary material for: Exploring the influence of electoral political orientation on health outcomes: a scoping review
Source: Cad Saude Publica. 2026 Apr 27;42:e00051525. doi: 10.1590/0102-311XEN051525 (PMC13170115; doi:10.1590/0102-311XEN051525)
Supplement: Supplementary Material [file 1678-4464-csp-42-EN051525-s.pdf]

SUPPLEMENTARY MATERIAL

Appendix 1 Search strategy.

1. PubMed/MEDLINE

13/12/2023 Pilot

(("electoral behavior" OR "electoral pattern" OR "electoral patterns" OR "Voting behavior" OR "Election influence" OR "Political trust" OR "Election choices" OR "Voter preferences" OR "Political participation" OR "Voting patterns" OR "Election decisions" OR "Political orientation" OR "Political identity" OR "Political beliefs" OR "Ideological affiliation" OR "Political ideology" OR "political views" OR "Political affiliation" OR "Party preferences" OR "Left-wing" OR "Right-wing" OR "Political spectrum" OR "Political values") AND ("Quality Indicators, Health Care" OR "Health Care Quality Indicators" OR "Health Status Indicators" OR "Health Indicators" OR "Health Indicator" OR "Health index" OR "Health Status Index" OR "Health Status Indexes" OR "Health Status Indices" OR "Health Metric" OR "Health Metrics"))

Number of results:65

https://pubmed.ncbi.nlm.nih.gov/?term=%28%28%22electoral+behavior%22+OR+%22electoral+pattern%22+OR+%22electoral+patterns%22+OR+%22Voting+behavior%22+OR+%22Election+influence%22+OR+%22Political+trust%22+OR+%22Election+choices%22+OR+%22Voter+preferences%22+OR+%22Political+participation%22+OR+%22Voting+patterns%22+OR+%22Election+decisions%22+OR+%22Political+orientation%22+OR+%22Political+identity%22+OR+%22Political+beliefs%22+OR+%22Ideological+affiliation%22+OR+%22Political+ideology%22+OR+%22political+views%22+OR+%22Political+affiliation%22+OR+%22Party+preferences%22+OR+%22Left-wing%22+OR+%22Right-wing%22+OR+%22Political+spectrum%22+OR+%22Political+values%22%29+AND+%28%22Quality+Indicators%2C+Health+Care%22+OR+%22Health+Care+Quality+Indicators%22+OR+%22Health+Status+Indicators%22+OR+%22Health+Indicators%22+OR+%22Health+Indicator%22+OR+%22Health+index%22+OR+%22Health+Status+Index%22+OR+%22Health+Status+Indexes%22+OR+%22Health+Status+Indices%22+OR+%22Health+Metric%22+OR+%22Health+Metrics%22%29%29&filter=lang.english&filter=lang.portuguese&filter=lang.spanish&filter=years.2014-2023

15/02/2024

(("electoral behavior" OR "electoral pattern" OR "electoral patterns" OR "Voting behavior" OR "Election influence" OR "Political trust" OR "Election choices" OR "Voter preferences" OR "Political participation" OR "Voting patterns" OR "Election decisions" OR "Political orientation" OR "Political identity" OR "Political beliefs" OR "Ideological affiliation" OR "Political ideology" OR "political views" OR "Political affiliation" OR "Party preferences" OR "Left-wing" OR "Right-wing" OR "Political spectrum" OR "Political values") AND ("Quality Indicators, Health Care" OR "Health Care Quality Indicators" OR "Health Status Indicators" OR "Health Indicators" OR "Health Indicator" OR "Health index" OR "Health Status Index" OR "Health Status Indexes" OR "Health Status Indices" OR "Health Metric" OR "Health Metrics"))

https://pubmed.ncbi.nlm.nih.gov/?term=%28%28%22electoral+behavior%22+OR+%22electoral+pattern%22+OR+%22electoral+patterns%22+OR+%22Voting+behavior%22+OR+%22Election+influence%22+OR+%22Political+trust%22+OR+%22Election+choices%22+OR+%22Voter+preferences%22+OR+%22Political+participation%22+OR+%22Voting+patterns%22+OR+%22Election+decisions%22+OR+%22Political+orientation%22+OR+%22Political+identity%22+OR+%22Political+beliefs%22+OR+%22Ideological+affiliation%22+OR+%22Political+ideology%22+OR+%22political+views%22+OR+%22Political+affiliation%22+OR+%22Party+preferences%22+OR+%22Left-wing%22+OR+%22Right-wing%22+OR+%22Political+spectrum%22+OR+%22Political+values%22%29+AND+%28%22Quality+Indicators%2C+Health+Care%22+OR+%22Health+Care+Quality+Indicators%22+OR+%22Health+Status+Indicators%22+OR+%22Health+Indicators%22+OR+%22Health+Indicator%22+OR+%22Health+index%22+OR+%22Health+Status+Index%22+OR+%22Health+Status+Indexes%22+OR+%22Health+Status+Indices%22+OR+%22Health+Metric%22+OR+%22Health+Metrics%22%29%29&filter=years.2000-2024

Number of results: 137

Updated: 28/02/2025

(("electoral behavior" OR "electoral pattern" OR "electoral patterns" OR "Voting behavior" OR "Election influence" OR "Political trust" OR "Election choices" OR "Voter preferences" OR "Political participation" OR "Voting patterns" OR "Election decisions" OR "Political orientation" OR "Political identity" OR "Political beliefs" OR "Ideological affiliation" OR "Political ideology" OR "political views" OR "Political affiliation" OR "Party preferences" OR "Left-wing" OR "Right-wing" OR "Political spectrum" OR "Political values") AND ("Quality Indicators, Health Care" OR "Health Care Quality Indicators" OR "Health Status Indicators" OR "Health Indicators" OR "Health Indicator" OR "Health index" OR "Health Status Index" OR "Health Status Indexes" OR "Health Status Indices" OR "Health Metric" OR "Health Metrics"))

Number of results: 10 – added to previous results and included in the main review up for revision.

2. Embase (Elsevier)

13/12/2023 Pilot

(("electoral behavior" OR "electoral pattern" OR "electoral patterns" OR "Voting behavior" OR "Election influence" OR "Political trust" OR "Election choices" OR "Voter preferences" OR "Political participation" OR "Voting patterns" OR "Election decisions" OR "Political orientation" OR "Political identity" OR "Political beliefs" OR "Ideological affiliation" OR "Political ideology" OR "political views" OR "Political affiliation" OR "Party preferences" OR "Left-wing" OR "Right-wing" OR "Political spectrum" OR "Political values") AND ("Quality Indicators, Health Care" OR "Health Care Quality Indicators" OR "Health Status Indicators" OR "Health Indicators" OR "Health Indicator" OR "Health index" OR "Health Status Index" OR "Health Status Indexes" OR "Health Status Indices" OR "Health Metric" OR "Health Metrics"))

Number of results:23.

15/02/2024

(("electoral behavior" OR "electoral pattern" OR "electoral patterns" OR "Voting behavior" OR "Election influence" OR "Political trust" OR "Election choices" OR "Voter preferences" OR "Political participation" OR "Voting patterns" OR "Election decisions" OR "Political orientation" OR "Political identity" OR "Political beliefs" OR "Ideological affiliation" OR "Political ideology" OR "political views" OR "Political affiliation" OR "Party preferences" OR "Left-wing" OR "Right-wing" OR "Political spectrum" OR "Political values") AND ("Quality Indicators, Health Care" OR "Health Care Quality Indicators" OR "Health Status Indicators" OR "Health Indicators" OR "Health Indicator" OR "Health index" OR "Health Status Index" OR "Health Status Indexes" OR "Health Status Indices" OR "Health Metric" OR "Health Metrics"))

Number of results: 40.

Updated: 28/02/2025

(("electoral behavior" OR "electoral pattern" OR "electoral patterns" OR "Voting behavior" OR "Election influence" OR "Political trust" OR "Election choices" OR "Voter preferences" OR "Political participation" OR "Voting patterns" OR "Election decisions" OR "Political orientation" OR "Political identity" OR "Political beliefs" OR "Ideological affiliation" OR "Political ideology" OR "political views" OR "Political affiliation" OR "Party preferences" OR "Left-wing" OR "Right-wing" OR "Political spectrum" OR "Political values") AND ("Quality Indicators, Health Care" OR "Health Care Quality Indicators" OR "Health Status Indicators" OR "Health Indicators" OR "Health Indicator" OR "Health index" OR "Health Status Index" OR "Health Status Indexes" OR "Health Status Indices" OR "Health Metric" OR "Health Metrics"))

Number of results: 3 – added to previous results and included in the main review up for revision.

3. CINAHL (EBSCO)

13/12/2022 Pilot

(("electoral behavior" OR "electoral pattern" OR "electoral patterns" OR "Voting behavior" OR "Election influence" OR "Political trust" OR "Election choices" OR "Voter preferences" OR "Political participation" OR "Voting patterns" OR "Election decisions" OR "Political orientation" OR "Political identity" OR "Political beliefs" OR "Ideological affiliation" OR "Political ideology" OR "political views" OR "Political affiliation" OR "Party preferences" OR "Left-wing" OR "Right-wing" OR "Political spectrum" OR "Political values") AND ("Quality Indicators, Health Care" OR "Health Care Quality Indicators" OR "Health Status Indicators" OR "Health Indicators" OR "Health Indicator" OR "Health index" OR "Health Status Index" OR "Health Status Indexes" OR "Health Status Indices" OR "Health Metric" OR "Health Metrics"))

Number of results: 6

15/02/2024

(("electoral behavior" OR "electoral pattern" OR "electoral patterns" OR "Voting behavior" OR "Election influence" OR "Political trust" OR "Election choices" OR "Voter preferences" OR "Political participation" OR "Voting patterns" OR "Election decisions" OR "Political orientation" OR "Political identity" OR "Political beliefs" OR "Ideological affiliation" OR "Political ideology" OR "political views" OR "Political affiliation" OR "Party preferences" OR "Left-wing" OR "Right-wing" OR "Political spectrum" OR "Political values") AND ("Quality Indicators, Health Care" OR "Health Care Quality Indicators" OR "Health Status Indicators" OR "Health Indicators" OR "Health Indicator" OR "Health index" OR "Health Status Index" OR "Health Status Indexes" OR "Health Status Indices" OR "Health Metric" OR "Health Metrics"))

Number of results: 18

Updated: 28/02/2025

(("electoral behavior" OR "electoral pattern" OR "electoral patterns" OR "Voting behavior" OR "Election influence" OR "Political trust" OR "Election choices" OR "Voter preferences" OR "Political participation" OR "Voting patterns" OR "Election decisions" OR "Political orientation" OR "Political identity" OR "Political beliefs" OR "Ideological affiliation" OR "Political ideology" OR "political views" OR "Political affiliation" OR "Party preferences" OR "Left-wing" OR "Right-wing" OR "Political spectrum" OR "Political values") AND ("Quality Indicators, Health Care" OR "Health Care Quality Indicators" OR "Health Status Indicators" OR "Health Indicators" OR "Health

Indicator" OR "Health index" OR "Health Status Index" OR "Health Status Indexes" OR "Health Status Indices" OR "Health Metric" OR "Health Metrics"))
Number of results: 2 – added to previous results and included in the main review up for revision.

4. Scopus (Elsevier)

13/12/2023 Pilot
(("electoral behavior" OR "electoral pattern" OR "electoral patterns" OR "Voting behavior" OR "Election influence" OR "Political trust" OR "Election choices" OR "Voter preferences" OR "Political participation" OR "Voting patterns" OR "Election decisions" OR "Political orientation" OR "Political identity" OR "Political beliefs" OR "Ideological affiliation" OR "Political ideology" OR "political views" OR "Political affiliation" OR "Party preferences" OR "Left-wing" OR "Right-wing" OR "Political spectrum" OR "Political values") AND ("Quality Indicators, Health Care" OR "Health Care Quality Indicators" OR "Health Status Indicators" OR "Health Indicators" OR "Health Indicator" OR "Health index" OR "Health Status Index" OR "Health Status Indexes" OR "Health Status Indices" OR "Health Metric" OR "Health Metrics"))
Number of results: 350
15/02/2024
(("electoral behavior" OR "electoral pattern" OR "electoral patterns" OR "Voting behavior" OR "Election influence" OR "Political trust" OR "Election choices" OR "Voter preferences" OR "Political participation" OR "Voting patterns" OR "Election decisions" OR "Political orientation" OR "Political identity" OR "Political beliefs" OR "Ideological affiliation" OR "Political ideology" OR "political views" OR "Political affiliation" OR "Party preferences" OR "Left-wing" OR "Right-wing" OR "Political spectrum" OR "Political values") AND ("Quality Indicators, Health Care" OR "Health Care Quality Indicators" OR "Health Status Indicators" OR "Health Indicators" OR "Health Indicator" OR "Health index" OR "Health Status Index" OR "Health Status Indexes" OR "Health Status Indices" OR "Health Metric" OR "Health Metrics"))
Number of results: 503
Updated: 28/02/2025
(("electoral behavior" OR "electoral pattern" OR "electoral patterns" OR "Voting behavior" OR "Election influence" OR "Political trust" OR "Election choices" OR "Voter preferences" OR "Political participation" OR "Voting patterns" OR "Election decisions" OR "Political orientation" OR "Political identity" OR "Political beliefs" OR "Ideological affiliation" OR "Political ideology" OR "political views" OR "Political affiliation" OR "Party preferences" OR "Left-wing" OR "Right-wing" OR "Political spectrum" OR "Political values") AND ("Quality Indicators, Health Care" OR

"Health Care Quality Indicators" OR "Health Status Indicators" OR "Health Indicators" OR "Health Indicator" OR "Health index" OR "Health Status Index" OR "Health Status Indexes" OR "Health Status Indices" OR "Health Metric" OR "Health Metrics"))
Number of results: 80 added to previous results and included in the main review up for revision.

5. Web of Science (Clarivate Analytics)

13/12/2023 Pilot
(("electoral behavior" OR "electoral pattern" OR "electoral patterns" OR "Voting behavior" OR "Election influence" OR "Political trust" OR "Election choices" OR "Voter preferences" OR "Political participation" OR "Voting patterns" OR "Election decisions" OR "Political orientation" OR "Political identity" OR "Political beliefs" OR "Ideological affiliation" OR "Political ideology" OR "political views" OR "Political affiliation" OR "Party preferences" OR "Left-wing" OR "Right-wing" OR "Political spectrum" OR "Political values") AND ("Quality Indicators, Health Care" OR "Health Care Quality Indicators" OR "Health Status Indicators" OR "Health Indicators" OR "Health Indicator" OR "Health index" OR "Health Status Index" OR "Health Status Indexes" OR "Health Status Indices" OR "Health Metric" OR "Health Metrics"))
Number of results: 15
15/02/2024
(("electoral behavior" OR "electoral pattern" OR "electoral patterns" OR "Voting behavior" OR "Election influence" OR "Political trust" OR "Election choices" OR "Voter preferences" OR "Political participation" OR "Voting patterns" OR "Election decisions" OR "Political orientation" OR "Political identity" OR "Political beliefs" OR "Ideological affiliation" OR "Political ideology" OR "political views" OR "Political affiliation" OR "Party preferences" OR "Left-wing" OR "Right-wing" OR "Political spectrum" OR "Political values") AND ("Quality Indicators, Health Care" OR "Health Care Quality Indicators" OR "Health Status Indicators" OR "Health Indicators" OR "Health Indicator" OR "Health index" OR "Health Status Index" OR "Health Status Indexes" OR "Health Status Indices" OR "Health Metric" OR "Health Metrics"))
Number of results: 19
Updated: 28/02/2025
(("electoral behavior" OR "electoral pattern" OR "electoral patterns" OR "Voting behavior" OR "Election influence" OR "Political trust" OR "Election choices" OR "Voter preferences" OR "Political participation" OR "Voting patterns" OR "Election decisions" OR "Political orientation" OR "Political identity" OR "Political beliefs" OR "Ideological affiliation" OR "Political ideology" OR "political views" OR "Political affiliation" OR "Party preferences" OR "Left-wing" OR "Right-

wing" OR "Political spectrum" OR "Political values") AND ("Quality Indicators, Health Care" OR "Health Care Quality Indicators" OR "Health Status Indicators" OR "Health Indicators" OR "Health Indicator" OR "Health index" OR "Health Status Index" OR "Health Status Indexes" OR "Health Status Indices" OR "Health Metric" OR "Health Metrics"))
Number of results: 3 added to previous results and included in the main review up for revision.

6. LILACS

13/12/2023 Pilot
(("comportamento eleitoral" OR "padrão eleitoral" OR "padrões eleitorais" OR "Comportamento de votação" OR "Influência eleitoral" OR "Escolhas eleitorais" OR "Preferências do eleitor" OR "Participação política" OR "Padrões de votação" OR "Decisões eleitorais" OR "Participação do eleitorado" OR "Orientação política" OR "Identidade política" OR "Crenças políticas" OR "Afiliação ideológica" OR "Identidade partidária" OR "Ideologia política" OR "visões políticas" OR "Filiação política" OR "Preferências partidárias" OR "extrema-direita" OR "Direita política" OR "extrema-Esquerda" OR "Esquerda política" OR "Espectro político" OR "Valores políticos" OR "comportamiento electoral" OR "patron electoral" OR "patrones electorales" OR "Comportamento de votación" OR "Influencia electoral" OR "Confianza política" OR "Preferencias del votante" OR "Participación política" OR "Patrones de votación" OR "Decisiones electorales" OR "Participación de votantes" OR "Orientación política" OR "Identidad política" OR "Creencias políticas" OR "Afiliación ideológica" OR "Identidad partidaria" OR "Ideologia política" OR "opiniones políticas" OR "Afiliación política" OR "Preferencias partidarias" OR "Izquierda política" OR "Extrema Izquierda" OR "derecha política" OR "Extrema derecha" OR "Espectro político" OR "electoral behavior" OR "electoral pattern" OR "electoral patterns" OR "Voting behavior" OR "Election influence" OR "Political trust" OR "Election choices" OR "Voter preferences" OR "Political participation" OR "Voting patterns" OR "Election decisions" OR "Political orientation" OR "Political identity" OR "Political beliefs" OR "Ideological affiliation" OR "Political ideology" OR "political views" OR "Political affiliation" OR "Party preferences" OR "Left-wing" OR "Right-wing" OR "Political spectrum" OR "Political values") AND ("Indicadores de Qualidade em Assistência à Saúde" OR "Indicadores Básicos de Saúde" OR "Indicadores de saúde" OR "Indicador de saúde" OR "índice de saúde" OR "Métrica de Saúde" OR "Métricas de Saúde" OR "Indicadores do Nível de Saúde" OR "Indicadores de Calidad de la Atención de Salud" OR "Indicadores de Salud" OR "Indicador de Salud" OR "Índice de Nivel de Salud" OR "Índice del Estado de Salud" OR "índice de salud" OR "Métrica Sanitaria" OR "Métrica de Salud" OR "Métricas Sanitarias" OR "Métricas de Salud" OR "Quality Indicators, Health Care" OR "Health

Care Quality Indicators" OR "Health Status Indicators" OR "Health Indicators" OR "Health Indicator" OR "Health index" OR "Health Status Index" OR "Health Status Indexes" OR "Health Status Indices" OR "Health Metric" OR "Health Metrics"))

Number of results:

LILACS: 1

CUMED: 1

MEDRxiv: 1

15/02/2024

Number of results:

Same as the pilot

Updated: 28/02/2025

(("comportamento eleitoral" OR "padrão eleitoral" OR "padrões eleitorais" OR "Comportamento de votação" OR "Influência eleitoral" OR "Escolhas eleitorais" OR "Preferências do eleitor" OR "Participação política" OR "Padrões de votação" OR "Decisões eleitorais" OR "Participação do eleitorado" OR "Orientação política" OR "Identidade política" OR "Crenças políticas" OR "Afiliação ideológica" OR "Identidade partidária" OR "Ideologia política" OR "visões políticas" OR "Filiação política" OR "Preferências partidárias" OR "extrema-direita" OR "Direita política" OR "extrema-Esquerda" OR "Esquerda política" OR "Espectro político" OR "Valores políticos" OR "comportamiento electoral" OR "patron electoral" OR "patrones electorales" OR "Comportamento de votación" OR "Influencia electoral" OR "Confianza política" OR "Preferencias del votante" OR "Participación política" OR "Patrones de votación" OR "Decisiones electorales" OR "Participación de votantes" OR "Orientación política" OR "Identidad política" OR "Creencias políticas" OR "Afiliación ideológica" OR "Identidad partidaria" OR "Ideologia política" OR "opiniones políticas" OR "Afiliación política" OR "Preferencias partidarias" OR "Izquierda política" OR "Extrema Izquierda" OR "derecha política" OR "Extrema derecha" OR "Espectro político" OR "electoral behavior" OR "electoral pattern" OR "electoral patterns" OR "Voting behavior" OR "Election influence" OR "Political trust" OR "Election choices" OR "Voter preferences" OR "Political participation" OR "Voting patterns" OR "Election decisions" OR "Political orientation" OR "Political identity" OR "Political beliefs" OR "Ideological affiliation" OR "Political ideology" OR "political views" OR "Political affiliation" OR "Party preferences" OR "Left-wing" OR "Right-wing" OR "Political spectrum" OR "Political values") AND ("Indicadores de Qualidade em Assistência à Saúde" OR "Indicadores Básicos de Saúde" OR "Indicadores de saúde" OR "Indicador de saúde" OR "índice de saúde" OR "Métrica de Saúde" OR "Métricas de Saúde" OR "Indicadores do Nível de Saúde" OR "Indicadores de Calidad de la Atención de Salud" OR "Indicadores de Salud" OR "Indicador de Salud" OR "Índice de Nivel de Salud" OR "Índice del

Estado de Salud" OR "índice de salud" OR "Métrica Sanitaria" OR "Métrica de Salud" OR "Métricas Sanitarias" OR "Métricas de Salud" OR "Quality Indicators, Health Care" OR "Health Care Quality Indicators" OR "Health Status Indicators" OR "Health Indicators" OR "Health Indicator" OR "Health index" OR "Health Status Index" OR "Health Status Indexes" OR "Health Status Indices" OR "Health Metric" OR "Health Metrics"))
Number of results: 0 added to previous results and included in the main review up for revision.

7. SciELO

13/12/2023 Pilot
(("comportamento eleitoral" OR "padrão eleitoral" OR "padrões eleitorais" OR "Comportamento de votação" OR "Influência eleitoral" OR "Escolhas eleitorais" OR "Preferências do eleitor" OR "Participação política" OR "Padrões de votação" OR "Decisões eleitorais" OR "Participação do eleitorado" OR "Orientação política" OR "Identidade política" OR "Crenças políticas" OR "Afiliação ideológica" OR "Identidade partidária" OR "Ideologia política" OR "visões políticas" OR "Filiação política" OR "Preferências partidárias" OR "extrema-direita" OR "Direita política" OR "extrema-Esquerda" OR "Esquerda política" OR "Espectro político" OR "Valores políticos" OR "comportamiento electoral" OR "patron electoral" OR "patrones electorales" OR "Comportamento de votación" OR "Influencia electoral" OR "Confianza política" OR "Preferencias del votante" OR "Participación política" OR "Patrones de votación" OR "Decisiones electorales" OR "Participación de votantes" OR "Orientación política" OR "Identidad política" OR "Creencias políticas" OR "Afiliación ideológica" OR "Identidad partidaria" OR "Ideologia política" OR "opiniones políticas" OR "Afiliación política" OR "Preferencias partidarias" OR "Izquierda política" OR "Extrema Izquierda" OR "derecha política" OR "Extrema derecha" OR "Espectro político" OR "electoral behavior" OR "electoral pattern" OR "electoral patterns" OR "Voting behavior" OR "Election influence" OR "Political trust" OR "Election choices" OR "Voter preferences" OR "Political participation" OR "Voting patterns" OR "Election decisions" OR "Political orientation" OR "Political identity" OR "Political beliefs" OR "Ideological affiliation" OR "Political ideology" OR "political views" OR "Political affiliation" OR "Party preferences" OR "Left-wing" OR "Right-wing" OR "Political spectrum" OR "Political values") AND ("Indicadores de Qualidade em Assistência à Saúde" OR "Indicadores Básicos de Saúde" OR "Indicadores de saúde" OR "Indicador de saúde" OR "índice de saúde" OR "Métrica de Saúde" OR "Métricas de Saúde" OR "Indicadores do Nível de Saúde" OR "Indicadores de Calidad de la Atención de Salud" OR "Indicadores de Salud" OR "Indicador de Salud" OR "Índice de Nivel de Salud" OR "Índice del Estado de Salud" OR "índice de salud" OR "Métrica Sanitaria" OR "Métrica de Salud" OR

"Métricas Sanitarias" OR "Métricas de Salud" OR "Quality Indicators, Health Care" OR "Health Care Quality Indicators" OR "Health Status Indicators" OR "Health Indicators" OR "Health Indicator" OR "Health index" OR "Health Status Index" OR "Health Status Indexes" OR "Health Status Indices" OR "Health Metric" OR "Health Metrics"))

Number of results: 0

15/02/2024

Number of results: Same as the pilot

Updated: 28/02/2025

Number of results: Same as the pilot 0

8. Academic Search Premier/ SocINDEX with Full Text(EBSCO)

13/12/2023 Pilot

(("electoral behavior" OR "electoral pattern" OR "electoral patterns" OR "Voting behavior" OR "Election influence" OR "Political trust" OR "Election choices" OR "Voter preferences" OR "Political participation" OR "Voting patterns" OR "Election decisions" OR "Political orientation" OR "Political identity" OR "Political beliefs" OR "Ideological affiliation" OR "Political ideology" OR "political views" OR "Political affiliation" OR "Party preferences" OR "Left-wing" OR "Right-wing" OR "Political spectrum" OR "Political values") AND ("Quality Indicators, Health Care" OR "Health Care Quality Indicators" OR "Health Status Indicators" OR "Health Indicators" OR "Health Indicator" OR "Health index" OR "Health Status Index" OR "Health Status Indexes" OR "Health Status Indices" OR "Health Metric" OR "Health Metrics"))

Number of results Academic Search Premier: 81

Number of results SocINDEX with Full Text: 20

15/02/2024

(("electoral behavior" OR "electoral pattern" OR "electoral patterns" OR "Voting behavior" OR "Election influence" OR "Political trust" OR "Election choices" OR "Voter preferences" OR "Political participation" OR "Voting patterns" OR "Election decisions" OR "Political orientation" OR "Political identity" OR "Political beliefs" OR "Ideological affiliation" OR "Political ideology" OR "political views" OR "Political affiliation" OR "Party preferences" OR "Left-wing" OR "Right-wing" OR "Political spectrum" OR "Political values") AND ("Quality Indicators, Health Care" OR "Health Care Quality Indicators" OR "Health Status Indicators" OR "Health Indicators" OR "Health Indicator" OR "Health index" OR "Health Status Index" OR "Health Status Indexes" OR "Health Status Indices" OR "Health Metric" OR "Health Metrics"))

Number of results Academic Search 101:

Number of results SocIndex 28:

Updated: 28/02/2025

(("electoral behavior" OR "electoral pattern" OR "electoral patterns" OR "Voting behavior" OR "Election influence" OR "Political trust" OR "Election choices" OR "Voter preferences" OR "Political participation" OR "Voting patterns" OR "Election decisions" OR "Political orientation" OR "Political identity" OR "Political beliefs" OR "Ideological affiliation" OR "Political ideology" OR "political views" OR "Political affiliation" OR "Party preferences" OR "Left-wing" OR "Right-wing" OR "Political spectrum" OR "Political values") AND ("Quality Indicators, Health Care" OR "Health Care Quality Indicators" OR "Health Status Indicators" OR "Health Indicators" OR "Health Indicator" OR "Health index" OR "Health Status Index" OR "Health Status Indexes" OR "Health Status Indices" OR "Health Metric" OR "Health Metrics"))

Number of results Academic Search 13: added to previous results and included in the main review up for revision.

Number of results SocIndex 4: added to previous results and included in the main review up for revision.

9. ProQuest Dissertations & Theses Global (PQDT Global)

13/12/2023 Pilot

NOFT(("electoral behavior" OR "electoral pattern" OR "electoral patterns" OR "Voting behavior" OR "Election influence" OR "Political trust" OR "Election choices" OR "Voter preferences" OR "Political participation" OR "Voting patterns" OR "Election decisions" OR "Political orientation" OR "Political identity" OR "Political beliefs" OR "Ideological affiliation" OR "Political ideology" OR "political views" OR "Political affiliation" OR "Party preferences" OR "Left-wing" OR "Right-wing" OR "Political spectrum" OR "Political values") AND ("Quality Indicators, Health Care" OR "Health Care Quality Indicators" OR "Health Status Indicators" OR "Health Indicators" OR "Health Indicator" OR "Health index" OR "Health Status Index" OR "Health Status Indexes" OR "Health Status Indices" OR "Health Metric" OR "Health Metrics"))

Number of results: 0

15/02/2024

Number of results: same as the pilot

Updated: 28/02/2025

Number of results: Same as the pilot 0

10. Theses & dissertations catalog CAPES

eleitor* AND indicador* AND saúde

Number of results: 0 for pilot and **15/02/2024**

Updated: 28/02/2025

Number of results: 0

11. Brazilian digital library of theses and dissertations (BDTD)

(("comportamento eleitoral" OR "padrão eleitoral" OR "padrões eleitorais" OR "Comportamento de votação" OR "Influência eleitoral" OR "Escolhas eleitorais" OR "Preferências do eleitor" OR "Participação política" OR "Padrões de votação" OR "Decisões eleitorais" OR "Participação do eleitorado" OR "Orientação política" OR "Identidade política" OR "Crenças políticas" OR "Afiliação ideológica" OR "Identidade partidária" OR "Ideologia política" OR "visões políticas" OR "Filiação política" OR "Preferências partidárias" OR "extrema-direita" OR "Direita política" OR "extrema-Esquerda" OR "Esquerda política" OR "Espectro político" OR "Valores políticos" OR "comportamiento electoral" OR "patron electoral" OR "patrones electorales" OR "Comportamento de votación" OR "Influencia electoral" OR "Confianza política" OR "Preferencias del votante" OR "Participación política" OR "Patrones de votación" OR "Decisiones electorales" OR "Participación de votantes" OR "Orientación política" OR "Identidad política" OR "Creencias políticas" OR "Afiliación ideológica" OR "Identidad partidaria" OR "Ideologia política" OR "opiniones políticas" OR "Afiliación política" OR "Preferencias partidarias" OR "Izquierda política" OR "Extrema Izquierda" OR "derecha política" OR "Extrema derecha" OR "Espectro político" OR "electoral behavior" OR "electoral pattern" OR "electoral patterns" OR "Voting behavior" OR "Election influence" OR "Political trust" OR "Election choices" OR "Voter preferences" OR "Political participation" OR "Voting patterns" OR "Election decisions" OR "Political orientation" OR "Political identity" OR "Political beliefs" OR "Ideological affiliation" OR "Political ideology" OR "political views" OR "Political affiliation" OR "Party preferences" OR "Left-wing" OR "Right-wing" OR "Political spectrum" OR "Political values") AND ("Indicadores de Qualidade em Assistência à Saúde" OR "Indicadores Básicos de Saúde" OR "Indicadores de saúde" OR "Indicador de saúde" OR "índice de saúde" OR "Métrica de Saúde" OR "Métricas de Saúde" OR "Indicadores do Nível de Saúde" OR "Indicadores de Calidad de la Atención de Salud" OR "Indicadores de Salud" OR "Indicador de Salud" OR "Índice de Nivel de Salud" OR "Índice del Estado de Salud" OR "índice de salud" OR "Métrica Sanitaria" OR "Métrica de Salud" OR "Métricas Sanitarias" OR "Métricas de Salud" OR "Quality Indicators, Health Care" OR "Health Care Quality Indicators" OR "Health Status Indicators" OR "Health Indicators" OR "Health

Indicator" OR "Health index" OR "Health Status Index" OR "Health Status Indexes" OR "Health Status Indices" OR "Health Metric" OR "Health Metrics"))

Number of results: 2 for pilot and **15/02/2024**

Updated: 28/02/2025

(("comportamento eleitoral" OR "padrão eleitoral" OR "padrões eleitorais" OR "Comportamento de votação" OR "Influência eleitoral" OR "Escolhas eleitorais" OR "Preferências do eleitor" OR "Participação política" OR "Padrões de votação" OR "Decisões eleitorais" OR "Participação do eleitorado" OR "Orientação política" OR "Identidade política" OR "Crenças políticas" OR "Afiliação ideológica" OR "Identidade partidária" OR "Ideologia política" OR "visões políticas" OR "Filiação política" OR "Preferências partidárias" OR "extrema-direita" OR "Direita política" OR "extrema-Esquerda" OR "Esquerda política" OR "Espectro político" OR "Valores políticos" OR "comportamiento electoral" OR "patron electoral" OR "patrones electorales" OR "Comportamento de votación" OR "Influencia electoral" OR "Confianza política" OR "Preferencias del votante" OR "Participación política" OR "Patrones de votación" OR "Decisiones electorales" OR "Participación de votantes" OR "Orientación política" OR "Identidad política" OR "Creencias políticas" OR "Afiliación ideológica" OR "Identidad partidaria" OR "Ideologia política" OR "opiniones políticas" OR "Afiliación política" OR "Preferencias partidarias" OR "Izquierda política" OR "Extrema Izquierda" OR "derecha política" OR "Extrema derecha" OR "Espectro político" OR "electoral behavior" OR "electoral pattern" OR "electoral patterns" OR "Voting behavior" OR "Election influence" OR "Political trust" OR "Election choices" OR "Voter preferences" OR "Political participation" OR "Voting patterns" OR "Election decisions" OR "Political orientation" OR "Political identity" OR "Political beliefs" OR "Ideological affiliation" OR "Political ideology" OR "political views" OR "Political affiliation" OR "Party preferences" OR "Left-wing" OR "Right-wing" OR "Political spectrum" OR "Political values") AND ("Indicadores de Qualidade em Assistência à Saúde" OR "Indicadores Básicos de Saúde" OR "Indicadores de saúde" OR "Indicador de saúde" OR "índice de saúde" OR "Métrica de Saúde" OR "Métricas de Saúde" OR "Indicadores do Nível de Saúde" OR "Indicadores de Calidad de la Atención de Salud" OR "Indicadores de Salud" OR "Indicador de Salud" OR "Índice de Nivel de Salud" OR "Índice del Estado de Salud" OR "índice de salud" OR "Métrica Sanitaria" OR "Métrica de Salud" OR "Métricas Sanitarias" OR "Métricas de Salud" OR "Quality Indicators, Health Care" OR "Health Care Quality Indicators" OR "Health Status Indicators" OR "Health Indicators" OR "Health Indicator" OR "Health index" OR "Health Status Index" OR "Health Status Indexes" OR "Health Status Indices" OR "Health Metric" OR "Health Metrics"))

Number of results: 0 added to previous results and included in the main review up for revision.

12. Google Scholar

13/12/2023 Pilot

("comportamento eleitoral" OR "Comportamento de votação" OR "Orientação política" OR "Identidade política" OR "extrema-direita" OR "extrema-Esquerda") AND ("Indicadores Básicos de Saúde" OR "Indicadores de saúde" OR "Indicador de saúde" OR "Métrica de Saúde" OR "Métricas de Saúde")

Number of results: 510

("comportamiento electoral" OR "Comportamento de votación" OR "Orientación política" OR "Identidad política" OR "Extrema Izquierda" OR "Extrema derecha") AND ("Indicadores de Salud" OR "Indicador de Salud" OR "Métrica de Salud" OR "Métricas Sanitarias" OR "Métricas de Salud")

Number of results:231

("electoral behavior" OR "Voting behavior" OR "Political orientation" OR "Political identity" OR "Left-wing" OR "Right-wing") AND ("Health Status Indicators" OR "Health Indicators" OR "Health Indicator" OR "Health Metric" OR "Health Metrics")

Number of results:2.550

15/02/2024

("comportamento eleitoral" OR "Comportamento de votação" OR "Orientação política" OR "Identidade política" OR "extrema-direita" OR "extrema-Esquerda") AND ("Indicadores Básicos de Saúde" OR "Indicadores de saúde" OR "Indicador de saúde" OR "Métrica de Saúde" OR "Métricas de Saúde")

Number of results: 776

("comportamiento electoral" OR "Comportamento de votación" OR "Orientación política" OR "Identidad política" OR "Extrema Izquierda" OR "Extrema derecha") AND ("Indicadores de Salud" OR "Indicador de Salud" OR "Métrica de Salud" OR "Métricas Sanitarias" OR "Métricas de Salud")

Number of results: 371

("electoral behavior" OR "Voting behavior" OR "Political orientation" OR "Political identity" OR "Left-wing" OR "Right-wing") AND ("Health Status Indicators" OR "Health Indicators" OR "Health Indicator" OR "Health Metric" OR "Health Metrics")

Number of results: 4020

Updated: 28/02/2025

("comportamento eleitoral" OR "Comportamento de votação" OR "Orientação política" OR "Identidade política" OR "extrema-direita" OR "extrema-Esquerda") AND ("Indicadores Básicos de

Saúde" OR "Indicadores de saúde" OR "Indicador de saúde" OR "Métrica de Saúde" OR "Métricas de Saúde")

Number of results: 62

("comportamiento electoral" OR "Comportamento de votación" OR "Orientación política" OR "Identidad política" OR "Extrema Izquierda" OR "Extrema derecha") AND ("Indicadores de Salud" OR "Indicador de Salud" OR "Métrica de Salud" OR "Métricas Sanitarias" OR "Métricas de Salud")

Number of results: 21

("electoral behavior" OR "Voting behavior" OR "Political orientation" OR "Political identity" OR "Left-wing" OR "Right-wing") AND ("Health Status Indicators" OR "Health Indicators" OR "Health Indicator" OR "Health Metric" OR "Health Metrics")

Number of results: 389

Despite the plethora of results available on Google Scholar, only the first five pages were selected for review. This decision was made following a thorough examination of the articles retrieved, which revealed a lack of relevance to the topic under investigation. While Google Scholar provides access to a vast array of scholarly literature, limiting the review to the first five pages was deemed appropriate to ensure that only the most pertinent and directly related articles were considered. This approach was adopted to maintain the focus of the review on articles that offer meaningful insights and contribute substantively to the research objectives. By prioritizing relevance and quality over quantity, the review process aimed to optimize the selection of articles for inclusion in the study, thereby enhancing the rigor and validity of the research findings.

Box S1 Full characteristics of the articles selected and included in this article.

#	Author	Study Title	Purpose	Keywords	Year of publication	Year of Study	Category	Topic area	Name of the journal	Publication type	Type of assessment	Method	Key Features of the evaluated program	Key variables	Geographic location	Population evaluated	Main findings	Gaps	Confounders
1	Sheryl Strasser, Sarah Trimmer, Georgia Atlanta. (12)	A County-Level Analysis of the Relationship between Voter Behavior as a Proxy for Partisan Ideology, Income, and the Effects on Health Morbidity and Mortality Measures	To analyze the relationship between voter behavior, partisan ideology, income, and their impact on health morbidity and mortality measures at the county level.	•County Health Rankings •Political partisanship •Voter behavior •Health morbidity and mortality •Health disparities	2013	2008 and 2012 Presidential Elections	Relationship between voting/political orientation/behaviour and health indicators	Public Health, Political Science, Health Disparities	Giorgia State University	Thesis	Two-way between-subjects ANOVA tests	University of Wisconsin Population Health Institute's County Health Rankings data for analysis	Focus on the influence of political partisanship and economic factors on health outcomes, especially among lower-income groups.	Partisan voter index category, income quartiles, health indicators (years of productive life lost, poor mental health days, poor physical health days)	United States	Residents at the county level	Significant interactions between income quartiles and partisan categories on health indicators.	Need for more research and methodological refinements, particularly in categorizing county-level political dynamics.	Not specified
2	Rafael Carijó Vellozo Lucas. (13)	A ideologia dos prefeitos e os impactos na saúde pública, um estudo de regressão descontínua	Analyze the impact of the political ideology of mayors on health indicators and health expenditure in municipalities	•Economia da Saúde •Regressão Descontínua •Ideologia e Resultados Econômicos •Análise de	2019	Data from 2005 to 2016	Relationship between voting/political orientation/behaviour and health indicators	Public Health, Political Science	Universidade Federal do Rio de Janeiro	Thesis	Empirical analysis	Regression Discontinuity Design (RDD)	•Comparison of health outcomes in municipalities governed by left-wing versus center and right-wing	•Political orientation of mayors •Health spending per capita •Number of low birth weight births •Infant mortality rate	Brazil	Municipalities in Brazil	•Significant partisan effect only on PSF coverage •Left-wing municipalities having higher coverage. •No significant partisan effect on other health indicators.	•Limited to health indicators and spending	Not specified

				Impacto									mayors<br>•Evaluation of per capita health spending, low birth weight births, infant mortality, and PSF coverage	•PSF coverage					
3	Chiara Rinaldi, Marleen P.M. Bekker. (14)	A Scoping Review of Populist Radical Right Parties' Influence on Welfare Policy and its Implications for Population Health in Europe	Mapping the available empirical evidence regarding the influence of populist radical right (PRR) parties on welfare policy reforms and to understand how this relationship is mediated by political system characteristics in different countries.	•Populist Radical Right<br>•Welfare Policy<br>•Europe	2021	literature published from 2000 to 2019	Relationship between voting/political orientation/ behaviour and health indicators	Public Health, Political Science	International Journal of Health Policy and Management	Peer-reviewed journal research article	Qualitative content analysis	Scoping review methodology	•Examination of PRR parties' exclusionary welfare policies<br>•Welfare chauvinism, impact of PRR parties on welfare policy, political system characteristics<br>•Welfare policy reforms and public health	•PRR parties' ideologies and actions<br>•Welfare policy reforms<br>•Population health outcomes<br>•Political system characteristics	Europe	•Vulnerable population groups<br>•European populations affected by welfare policies	•PRR parties' welfare chauvinistic positions negatively affect the health of vulnerable population groups<br>•Differences in the implementation of welfare chauvinistic policies mediated by constitutional order and partianship<br>•Welfare chauvinism is more pronounced in countries with tax-based healthcare systems	•Limited empirical literature directly connecting PRR parties to health outcomes.<br>•Lack of comprehensive data on the role of interest representation of effects of policies	Variability in political system characteristics
4	S.V. Subraman	Are Republica	Investigate	•Political ideology	2010	Data from	Relationship	Public Health,	International Journal	Peer-review	Statistical	Weighted, binary	•Analysis of health	•Political ideology	United	Individuals	•Republicans report lower	The study does not	Socio-economic

	ian Jessica M. Perkins. (15)	ns Healthier than Democrats ?	whether there are fundamental differences in health status and behaviors between individuals who identify with conservative and liberal political parties in the USA.	•Health status •Smoking •Republicans •Democrats •USA		1972–2006	between voting/political orientation/ behaviour and health indicators	Political Science	of Epidemiology	ed journal research article	analysis of survey data	logistic regression model procedures	status and smoking status based on political ideology •Comparison between Republicans, Democrats, and Independents	•Health status •Smoking status	States	participating in the General Social Surveys (GSS)	rates of poor health and are less likely to be smokers compared to Democrats. •These differences persist even after adjusting for several covariates	establish causality between political ideology and health outcomes .	status (SES), religiosity, and potential inadequacies
5	Thomas J. Bollyky et.al. (16)	Assessing COVID-19 Pandemic Policies and Behaviours and Their Economic and Educational Trade-offs across US States from Jan 1, 2020, to July 31, 2022: An Observational Analysis	Identify factors associated with cross-state variation in COVID-19 infection and mortality rates in the US and assess the trade-offs between health outcomes and economic and educational impacts.	•COVID-19 •Pandemic •Policies •Behaviours •Economic trade-offs •Educational trade-offs •US states	2023	Jan 1, 2020, to July 31, 2022	Relationship between voting/political orientation/ behaviour and vacination	Public Health, Political Science	The Lancet	Peer-reviewed journal research article	Observational analysis	Regression Analysis, Data Standardization	•Analysis of state-level policies in relation to COVID-19 outcomes	•COVID-19 death rates •Infection rates •Policy mandates •Vaccination coverage •Mask use •Economic and educational trade-offs	United States	Population of US states	•Social, economic, and racial inequities were associated with higher COVID-19 infection •Political affiliation of state governors did not significantly affect outcomes •States with higher proportions of Republican voters had worse outcomes.	•The study did not fully explore individual-level factors and impact with COVID-19 •Limited by observational nature and potential unmeasured confounding factors.	Population density, age distribution, comorbidities, socio-economic status

6	James S. Goodwin et.al. (17)	Association of Chronic Opioid Use With Presidential Voting Patterns in US Counties in 2016	To explore the overlap between the geographic distribution of high opioid use in US counties and the vote for the Republican candidate in the 2016 presidential election, and to investigate how individual and county-level demographic and economic measures explain this association.	•Chronic opioid use •Presidential voting patterns •US counties •2016 election •Republican vote •Socioeconomic measures	2018	Data from 2015 (opioid use) and 2016 (voting data)	Relationship between voting/political orientation/ behaviour and health indicators	Public Health, Political Science	JAMA Network Open	Peer-reviewed journal research article	Cross-sectional analysis	Multilevel analysis	•Analysis of chronic opioid use by county rate •Correlation between county-level opioid use rates and the percentage of votes for the Republican candidate in 2016 •Consideration of individual and county-level socioeconomic measures	•County rate of chronic opioid use •Percentage of Republican presidential vote •Socioeconomic measures	United States	Medicare Part D enrollees	•Significant correlation (0.42, P < .001) between a county’s Republican presidential vote and the adjusted rate of prolonged opioid prescriptions. •Support for the Republican candidate is a marker for physical, economic associated with opioid use.	•The study is ecological and measures associations at the county level, not the individual level. •Limited to data from Medicare Part D enrollees •Characteristics of opioid prescribers not included.	Socioeconomic variables, county-level demographic characteristics, health status and comorbidities
7	Jessica L. Krok-Schoen et.al. (18)	Belief About Mandatory School Vaccinations and Vaccination Refusal	To examine how demographic, general health, religious,	•Adolescent immunization •Ohio Appalachia •Parents	2018	2013 and 2014	Relationship between voting/political orientation/ behaviou	Political Health, Social Determinants of Health	Journal of Rural Health	Peer-reviewed journal research article	Empirical research study	Multivariate logistic regression models	The study evaluates parental beliefs and behaviors	Political affiliation (Republican, Independent, Democrat)	Ohio Appalachia, USA	Parents (n = 337) of girls aged 9–17 from 12 counties in rural	•47% of parents believed they should have the right to refuse mandatory school vaccinations. •Political	•The study does not address reasons for vaccine refusal in	Possible confounders include socioeconomic status, access to healthcare, and

		Among Ohio Appalachian Parents	and political characteristics influenced beliefs about mandatory school vaccinations and history of vaccination refusal for children among Ohio Appalachian parents.	•Social determinants of health •Vaccines			r and vacination						regarding mandatory school vaccinations, specifically focusing on the influence of political affiliation, religiosity, general health, and demographic factors on vaccination refusal.	Gender Religious identity and religiosity General health status Education level Household income Employment status Marital status Smoking status		Ohio Appalachia	affiliation significantly influenced these beliefs, with Republicans and Independents more likely to support the right to refuse than Democrats. •39% of parents reported having refused a vaccine for their child, with significant predictors being female gender and the belief in the right to refuse school vaccinations.	detail. •Limited generalizability due to the specific regional focus on Ohio Appalachia. •Lack of data on specific vaccines refused and reasons for refusal.	cultural attitudes that were not fully controlled for in the analysis.
8	Saerom Kim Chang-yup Kim Myung Soon You. (19)	Civic Participation and Self-rated Health: A Cross-national Multi-level Analysis Using the World Value Survey	To examine the effect of civic participation on self-rated health status using a multi-level analysis of data from the World Value Survey across 44 countries	Participation Self-rated health Multilevel analysis World Value Survey	2015	2005-2008	Relationship between voting/political orientation/behaviour and health indicators	Public Health, Civic Participation	Journal of Preventive Medicine & Public Health	Peer-reviewed journal research article	Empirical research study	Multi-level logistic regression analysis	The study evaluates the relationship between various forms of civic participation (voting, conventional and unconventional political activities, social	Political participation (conventional and unconventional) Social participation Self-rated health Country-level variables	44 countries included in the World Value Survey	Individuals (n=50,859) from 44 countries who participated in the survey	•People who participated in voting and voluntary social activities reported better self-rated health. •Unconventional political participation was negatively associated with subjective health but became insignificant in OECD countries. •The democratic index and public health	•Cross-sectional design limits causal inferences. •Potential for reverse causality. •Reliance on self-rated health as the sole measure of health status. •Lack of	•Age, sex, marital status, education, and income were controlled at the individual level. •Economic status, health spending, and democratic index were controlled at the

													participation) and self-rated health status at both individual and country levels.				expenditure were significant factors in determining self-rated health. •Social participation had a contextual association with subjective health status, with variations between the full sample and OECD countries.	additional country-level determinants like social trust, income inequality, type of health care system, and welfare regime.	country level.
9	Agda Araujo da Nóbrega Marcia Lúcia Avila de Melo. (20)	Coalizão partidária - aporte financeiro - desempenho de assistência médica e odontologia na atenção básica no Brasil	To describe the policy orientation of party coalitions, budget outlay, and the structure and performance of medical and dental care	•Política de saúde •Financiamento público •Assistência médica •Assistência odontológica •Brasil	2018	Data from 2008 to 2013	Relationship between voting/political orientation/behaviour and health indicators	Public Health, Political Science	Cadernos de Saúde Pública	Peer-reviewed journal research article	Descriptive, retrospective case study	Regression analysis	•Examination of financial investments and political coalitions' impact on healthcare •Analysis of healthcare indicators such as service coverage, quality of care	•Political coalition •Financial investment •Performance outcomes in medical and dental care	Brazil	Municipalities in Brazil	•Political coalitions and financial investments significantly influence the performance of primary healthcare services. •Municipalities governed by coalitions showed better healthcare outcomes.	•Limited by the availability and quality of financial and performance data. •Other socio-political factors was not examined	Socio-economic conditions, Differences in healthcare infrastructure and resource availability.
10	Patrick Bernet. (21)	COVID-19 Infections and Mortality in Florida Counties: Roles of	Investigate the association of racial and ethnic composit	•COVID-19 •Racial and ethnic disparities •Segregat	2021	Data collected through March 2021	Relationship between voting/political orientation/behaviou	Public Health, Epidemiology, Political Science	Journal of Racial and Ethnic Health Disparities	Peer-reviewed journal research article	Ecological Study	Poisson regression analysis	•Higher COVID-19 infection and mortality rates in counties	•COVID-19 infection rates •COVID-19 mortality rates	Florida, USA	Population of Florida counties	•Higher proportions of Black residents experience disproportionately higher COVID-19 infection and	•The study focuses on county-level data, which	Socio-economic factors, demographic variations, healthcare infrastructu

		Race, Ethnicity, Segregation, and 2020 Election Results	ion, segregation, and 2020 presidential election voting results with COVID-19 infections and deaths in Florida counties.	ion •Political affiliation			r and vacination						with higher proportions of Black and Hispanic residents and larger Republican vote shares	•Racial and ethnic composition •Residential segregation •Political affiliation			mortality rates. •Disparities are further inflated in counties with larger Republican vote shares. •Hispanic population proportions and segregation are also associated with higher COVID-19 infection and mortality rates in more Republican-leaning counties.	may not capture individual-level factors •Limited generalizability beyond Florida	re
11	George B. Cunningham, Calvin Nite. (22)	Demographics, politics, and health factors predict mask wearing during the COVID-19 pandemic: a cross-sectional study	To examine the association between health determinants, demographics, and voting patterns on mask-wearing behavior at the county level in the United States during the COVID-19 pandemi	•COVID-19 •Mask wearing •Health behaviors •Political orientation	2021	Data collected in July 2020	Relationship between voting/political orientation/ behaviour and vacination	Public Health, Political Science	BMC Public Health	Peer-reviewed journal research article	Cross-sectional study	Two-level random effects regression models	•How health behaviors, clinical care, social and economic conditions, and the physical environment influence mask-wearing behavior. •County demographics and voting patterns serve as control variables	•Dependent variable: Percent of county residents frequently or always wearing a mask •Independent variables: Health behaviors, Clinical care, Social and economic conditions, Physical environment •Control	United States (county-level analysis)	County residents across the United States (n=3,142 counties)	•Health behaviors were positively associated with mask-wearing. •The physical environment was negatively associated with mask-wearing. •Clinical care and social and economic factors were not significantly associated with mask-wearing. •Counties with higher percentages of Democratic voters had higher rates of mask-wearing.	•Reliance on self-reported mask-wearing behavior at a single point in time. •Does not capture within-county variability. •Limited to county-level analysis in the United States, which may not	Controlled for age, gender, race, rural residence, and political persuasion.

			c.											variables: Age, gender, race, rural vs. urban residence, political persuasion (percent voting Democrat in 2016 election)				be applicable to other settings. •Did not consider psychological factors that might influence mask-wearing, such as prosociality and altruism.	
12	Jacob Bor. (23)	Diverging Life Expectancies and Voting Patterns in the 2016 US Presidential Election	To assess whether voting patterns in the 2016 US presidential election were correlated with long-term trends in county life expectancy.	•Life expectancy •Voting patterns •US presidential election •Political orientation	2017	Data from 1980 to 2014 for life expectancy, voting data from 2008 and 2016 elections	Relationship between voting/political orientation/ behaviour and health indicators	Public Health, Political Science	American Journal of Public Health	Peer-reviewed journal research article	Observational analysis	Multivariable regression analysis adjusting for county demographic and economic characteristics	Association between changes in county life expectancy from 1980 to 2014 and voting patterns in the 2008 and 2016 presidential elections.	•Life expectancy at birth •Trump's share of the 2016 vote •Change in the Republican vote	United States	County-level data across the United States	•Less suport for Trump from Counties experiencing greater gains in life	Limited by the use of aggregated county-level data	State, rural/metropolitan status, education level, income inequality, unemployment, median home value, poverty, economic mobility, racial/ethnic composition
13	Michal Hrivnák et.al. (24)	Does Civic Engagement Support Relational and Mental	To estimate the impact of civic engagement (both	•Community engagement •Political engagement	2023	Data collected in 2022	Relationship between voting/political orientation/	Public Health, Civic Engagement	Journal name: "Societies"	Peer-reviewed journal research article	Empirical study using econometric tools	Electronic survey and cross-sectional regression models	•Investigates the impact of different forms of civic engagem	•Dependent Variables: Relational health, mental health	Nitra, Slovakia	Residents of Nitra, Slovakia (n = 318)	•Positive effects of community engagement on relational health. •Political participation contributed to	•Relies on self-reported data, which may introduce	Socioeconomic factors, such as income and education,

		Health of Urban Population?	political and community engagement) on relational and mental health in a medium-sized urban settlement in Slovakia.	•Volunteerism<br>•Well-being<br>•Mental health<br>•Relational health			behaviour and health indicators						ent on health outcomes.<br>•Uses quantitative data from an urban area in Slovakia.	•Independent Variables: Political engagement, community engagement, volunteerism<br>•Control Variables: Age, gender, faith, family status, children, education, income, free time			the reduction of depressive symptoms, but no significant relationship was found between community engagement and mental health.<br>•High levels of political engagement negatively impacted relational health.	bias.<br>•Limited generalizability due to focus on a single medium-sized city in Slovakia.<br>•Does not capture the long-term effects of civic engagement on health.	which were controlled for in the analysis.
14	Sanghoon Lee. (25)	Does Democracy Matter for Public Health?	Empirically analyze the relationship between democracy and public health using robust econometric methods on panel data from 188 countries.	•Democracy<br>•Public health<br>•Panel data	2023	Data collected from 1972 to 2019	Relationship between voting/political orientation/behaviour and health indicators	Public Health, Political Science	International Journal of Social Determinants of Health and Health Services	Peer-reviewed journal research article	Empirical study using various regression methods	Pooled OLS, fixed effects, dynamic GMM, split-sample methods, and quadratic models	The study explores the direct and indirect effects of democracy on health outcomes, emphasizing historical and cumulative impacts.	•Dependent Variables: Life expectancy, infant mortality<br>•Independent Variables: Democracy (measured by Freedom House index)<br>•Control Variables: GDP per capita, Gini index,	Global (n=188 countries)	Country-level data from 188 countries	•Democracy positively impacts public health, especially in reducing infant mortality and increasing life expectancy.<br>•There is a threshold effect where the positive impact of democracy on health is less evident in low-income countries.<br>•A U-shaped relationship exists between democracy and infant mortality.	The effect of democracy on health outcomes is not uniform across different income levels, indicating a threshold effect.	•Economic performance and inequality, represented by GDP per capita and Gini index, respectively.<br>•Health infrastructure, measured by the number of physicians per 1,000 people.

														number of physicians per 1,000 people					
15	Manthos D. Delis, Maria Iosifidi, Menelaos Tasiou. (26)	Efficiency of government policy during the COVID-19 pandemic	Introduce new indices for measuring the efficiency of government policies in dealing with the COVID-19 pandemic and to identify the determinants of government policy efficiency.	•Frontier methods •Government efficiency •COVID-19 pandemic •Oxford COVID-19 Government •Response Tracker •Determinants of efficiency	2023	Data from May 2020 to November 2021	Relationship between voting/political orientation/ behaviour and vacination	Public Health, Political Science	Annals of Operations Research	Peer-reviewed journal research article	Empirical study using data envelopment analysis (DEA) and stochastic frontier analysis (SFA)	DEA and SFA were used to create government policy efficiency indices	•Analyzes the efficiency of government policies in minimizing COVID-related deaths. •Uses comprehensive data from multiple countries and robust econometric techniques.	•Dependent Variables: COVID-19-related deaths •Independent Variables: Government policy stringency Control Variables: Institutions, democratic principles, political stability, trust, public health spending, female participation in the workforce, economic equality	Global (n=81 countries)	Country-level data from 81 countries	Positive correlation between government policy efficiency and factors like institutional quality, democratic principles, political stability, and high public health spending. Negative correlation between economic inequality and government policy efficiency.	The study does not make causal claims, focusing instead on correlates of government policy efficiency.	GDP per capita, economic inequality, and health infrastructure
16	Sankar Mukhopadhyay. (27)	Elections have (health) consequences: Depression, anxiety,	To examine the effect of the 2020 presidential	•Depression •Anxiety •Presidential Election •	2022	Data from April 2020 to December 2021	Relationship between voting/political orientation/	Public Health, Political Science Mental health	Scientific Journal Article	Peer-reviewed journal research article	Cross-sectional analysis	Logistic regressions	•Analysis of self-reported symptoms of anxiety and	•Self-reported symptoms of moderate to severe anxiety	United States	2020 Household Pulse Survey (n=2.45 million)	•Moderate to severe anxiety and depression increased steadily up to the presidential election	•Did not account for underlying causes of the mental	COVID-19 activity Demographic and economic characteristics

		and the 2020 presidential election	election on anxiety and depression among Americans, using data from the 2020 Household Pulse Survey (HPS).	COVID-19 •Mental health			behaviour and mental health indicators						depression before and after elections •Use of the 2020 Household Pulse Survey (HPS) •Consideration of anxiety and depression-specific office visits and mental heath prescriptions	and depression •Mental health visits •Mental health prescription drug usage •Demographic and economic characteristics •State-level COVID-19 activity			•Those symptoms decreased afterwards. •moderate to severe anxiety and depression increased steadily up to the presidential election	health conditions. •The high non-response rate (23%) could potentially bias the results.	State-level differences
17	Helena Arruda, Rudi Rocha. (28)	Eleições Municipais e Transições de Governo: Disrupção de Serviços e Aumento da Mortalidade Infantil	This study examines the effects of municipal political transitions on the provision of health services and infant health indicators, particularly focusing on critical periods for human	•Eleições municipais •Eransições de governo •Mortalidade infantil •Disrupção de serviços •Saúde pública •Brasil	2024	Recent Brazilian municipal elections	Relationship between voting/political orientation/ behaviour and health indicators	Public Health, Political Science	Instituto de Estudos para Políticas de Saúde (IEPS)	Technical Report	Observational study	Causal inference methods	•Impact of government transitions on the provision of primary health services •Analysis of disruptions in health service provision •Investigates the effects on infant health	•Number of prenatal consultations •Infant birth weight •Infant mortality rates •Timing of government transition	Brazil	Municipalities in Brazil	•Political transitions lead to a temporary but significant reduction in health service provision •Infants exposed to these transitions have higher chances of being born with low birth weight •Infants exposed to these transitions have an increased infant mortality rates.	•Does not explore underlying causes of staff turnover in detail. •Limited by the assumption that electoral outcomes	Socioeconomic and demographic characteristics of municipalities

			development.										outcomes						
18	Jason Beckfield, Nancy Krieger. (29)	Epi + demos + cracy: Linking Political Systems and Priorities to the Magnitude of Health Inequities—Evidence, Gaps, and a Research Agenda	Review and synthesize empirical studies linking political systems and priorities to health inequities and propose a research agenda to address identified gaps.	•Democracy •Epidemiology •Health Status •Health Inequities •Politics •Public Health •Social Class •Socioeconomic Factors	2009	Reviewed studies from 1992 to 2008	Relationship between voting/political orientation/behaviour and health indicators	Public Health, Political Science	Epidemiologic Reviews	Peer-reviewed journal research article	Systematic review	Systematic search and review of studies using databases ISI Web of Knowledge and PubMed.	•Review of 45 studies on political determinants of health inequities. •Focus on transitions to capitalism, neoliberal restructuring, welfare states, and political incorporation of marginalized groups.	•Health inequities (magnitude and changes) •Political systems and policies	Global, with a focus on the global North.	Various populations across different countries	•Transition to capitalism and neoliberal restructuring likely increase health inequities. •Welfare states have mixed effects on health inequities. •Political incorporation of marginalized groups tends to reduce health inequities.	Limited focus on the global South and need for more comprehensive research on political determinants of health inequities.	Variability in political contexts, policy implementations, and socioeconomic factors
19	Arthur Chioro et.al. (30)	From Bolsonaro to Lula: The opportunity to rebuild universal healthcare in Brazil in the government transition	To analyze the transition from the Bolsonaro government to the Lula government in Brazil and its implications for	•Brazilian health system •Democracy •Global health policies •Health policy •Political challenges	2023	Government transition period between 2022 and 2023	Relationship between voting/political orientation/behaviour and health indicators	Public Health, Political Science	International Journal of Health Planning and Management	Peer-reviewed journal research article	Policy analysis	Case study	•Outlines the dismantling of the Brazilian national health system (SUS) under the Bolsonaro government •Highlig	•Political regime (Bolsonaro vs. Lula) •Health system funding •Health policy changes	Brazil	Population as a whole (SUS users)	•Bolsonaro administration's policies led to significant defunding and weakening of the SUS •Lula administration has the opportunity to rebuild the SUS, emphasizing the importance of universal health	•Descriptive study with no provide empirical data •Focuses on political and policy analysis rather than quantitati	Differences in regional implementation of health policies and socio-economic conditions.

			the Brazilian health system, focusing on the challenges and opportunities to rebuild and strengthen universal healthcare.										hts the efforts needed to rebuild it under the Lula administration. •Emphasizes the need for a renewed commitment to universal health coverage				coverage	ve health impact assessment.	
20	Laura Duggan. (31)	Gender and Political Orientation as Predictors of Prosocial Behaviour in Ireland	This study examines the influence of gender and political orientation on prosocial behavior	•Prosocial behavior •Gender •Political orientation •Ireland •Empathy •Social attitudes	2022	2022	Relationship between voting/political orientation/ behaviour and mental health indicators	Public Health, Political Science, Sociology	National College of Ireland	Thesis	Quantitative survey analysis	Hierarchical regression analyses	•Significant association between political orientation and prosocial behavior •Minor predictive link between gender and prosocial behavior.	•Gender •Political orientation •Prosocial behavior	Ireland	Irish adults (N=313)	•Left-wing individuals show higher prosocial behavior compared to right-wing individuals •Women exhibit higher prosocial behavior than men (emotional prosocial behavior)	•Limited to Ireland •Bias in self-report measures.	Gender and political orientation interactions
21	Tetsuya Matsubayashi Michiko Ueda. (32)	Government Partisanship and Human Well-Being	Examine the effect of government partisanship on citizens' well-	•Government partisanship •Happiness •Life satisfaction	2011	1980-2004	Relationship between voting/political orientation/ behaviour and	Public Health, Political Science	Springer Science+Business Media B.V.	Peer-reviewed journal research article	Empirical analysis using survey data and panel data	Panel data analysis	•Investigates the effects of left-leaning vs. right-leaning governments on	•Life satisfaction •Suicide rates •GDP growth rate •Unemplo	21 OECD countries	Citizens of 21 OECD countries	Left-leaning and Christian democratic governments are associated with higher life satisfaction and lower suicide rates.	•Limited to OECD countries •Potential cultural differences not fully accounte	Macroeconomic variables, government policies, socio-demographic characterist

			being, measured by life satisfaction and suicide rates.	•Subjective well-being •Welfare policy •Suicide			health indicators						well-being indicators.	yment rate •Inflation rate •Welfare spending •Social expenditure				d for	ics
22	Arjumand Siddiqi et.al. (33)	Growing Sense of Social Status Threat and Concomitant Deaths of Despair Among Whites	Investigate the rising mortality rates among whites in the United States, particularly focusing on the concept of "deaths of despair" linked to perceived social status threat rather than traditional economic factors	•Race •Mortality •Republican party •Voting •Social determinants of health	2019	2000-2016	Relationship between voting/political orientation/ behaviour and health indicators	Political Health, Social Determinants of Health	SSM - Population Health	Peer-reviewed journal research article	Empirical analysis using administrative and survey data	County-level fixed effects model	•Analysis of mortality trends among different educational and age groups of whites. •Examination of the relationship between perceived social status threat and rising mortality rates.	•Mortality rates •Education levels •Economic and social indicators •Voting patterns (Republican share)	United States	Non-Hispanic whites, aged 25-54	•Rising mortality among whites is not confined to the least educated •Perceptions of social status threat are linked to the increasing Republican voting share	•Limited exploration of the mechanisms linking perceived •The study does not account for potential protective factors among other racial groups	Economic conditions that affect both black and white, Variations in local social and policy environments.
23	Max E. Coleman Matthew A. Andersson. (34)	Hurt on Both Sides: Political Differences in Health and Well-Being during the	Investigate how political background (party, ideology, voting behavior,	•Mastery •Mental health •Political background •Self-rated health	2024	Data collected in early 2021	Relationship between voting/political orientation/ behaviour and	Public Health, Political Science	Journal of Health and Social Behavior	Peer-reviewed journal research article	Quantitative analysis of survey data.	Linear regression models.	Analysis of health outcomes such as self-rated health, psychological distress,	•Political background (party, •Ideology, voting behavior, policy beliefs) •Health	United States	A random sample of 11,000 U.S. households.	•Republicans report better self-rated health and less distress compared to Democrats. •Liberal beliefs are robust predictors of	Limits the ability to assess within-person changes over time.	Demographic variables, socioeconomic status, psychosocial resources.

		COVID-19 Pandemic	policy beliefs) predicts differences in health and well-being indicators during the COVID-19 pandemic	•Social support			health indicators						happiness, sleep disturbances, and delayed healthcare based on political affiliation and beliefs.	indicators •Psychosocial mechanisms (mastery, inequality beliefs, social support) •COVID-19 exposures and attitudes			health differences. •COVID-19 exposures and attitudes significantly mediate these political health differences.		
24	Diana J. Burgess et.al. (35)	Incoming Medical Students' Political Orientation Affects Outcomes Related to Care of Marginalized Groups	Characterize the political ideology of first-year medical students and to assess how their political ideology predicts attitudes and beliefs related to the care of marginalized patients by their fourth year.	•Medical education •Implicit bias •Stigmatization •Health care disparities	2019	2010 - 2014 Follow up	Relationship between voting/political orientation/ behaviour and health indicators	Public Health, Medical Education	Journal of Health Politics, Policy and Law	Peer-reviewed journal research article	Longitudinal study	Mixed-effects linear regression analysis of survey data	•Assessed political ideology on a five-point scale from "very conservative" to "very liberal." •Evaluated implicit and explicit biases, motivation to control prejudice, empathy, patient-centered orientation, and interracial anxiety.	•Political orientation •Implicit bias •Explicit prejudice •Motivation to control prejudice •Empathy (emotional and cognitive) •Patient-centered orientation Interracial anxiety	United States	Medical students from a stratified random sample of 49 medical schools	•Conservative political ideology in first-year students predicted higher levels of bias and negative attitudes towards marginalized groups in their fourth year. •Conservative students showed lower motivation to control prejudice and lower empathy.	•Did not examine how political ideology affects actual treatment outcomes. •The study focused on attitudes and beliefs, not behaviors.	Variability in curricula and experiences, Self-selection bias in survey participation.

25	C Kelleher, A Timoney, S Friel, D McKeown. (36)	Indicators of deprivation, voting patterns, and health status at area level in the Republic of Ireland	To determine the relationship between mortality patterns, indicators of deprivation, general lifestyle, and social attitudes, as exemplified by general election voting patterns.	•Voting Patterns •Mortality •Deprivation •Political Orientation •Health Status •Social Attitudes	2002	Data up to 1997	Relationship between voting/political orientation/ behaviour and health indicators	Public Health, Epidemiology, Political Science	Journal of Epidemiology and Community Health	Peer-reviewed journal research article	Cross-sectional ecological study	Correlation and regression analyses	•Analysis of voting data from the 1997 general election. •Calculation of standardized mortality ratios for different county areas.	•Voting patterns •Standardized mortality ratios •Indicators of deprivation •Health status indicators •Lifestyle factors	Ireland	Adults over 18 years (n=273 disctricts)	•Relations between left-wing voting and health dissatisfaction and smoking rates. •No significant relation between SMR and voting patterns for Fianna Fail and Fine Gael. •Fianna Fail voting pattern inversely related to dissatisfaction with health.	•Temporal relevance outside the specified timeframe. •Limited to cross-sectional data	Sociodemographic variables (not specified)
26	Salvatore Perri. (37)	Italy on the Way to Trumpism	To analyze the potential effects of implementing Matteo Salvini's economic program and political strategies on the Italian economy and society.	•Voting behavior •Economic policies •Italy •Salvinism •Trumpism	2019	Data and political events up to 2019.	Relationship between voting/political orientation/ behaviour and health indicators	Public Health, Political Science	International Journal of Political Economy	Peer-reviewed journal research article	Qualitative and analytical study.	Analysis of economic data, political strategies, communication methods, and electoral trends.	•Examination of Salvini's economic and political strategies. •Comparison of Salvini's methods with those of Donald Trump. •Analysis of potential socio-economic impacts.	•Political orientation •Economic policies •Socio-economic outcomes	Italy	Italian electorate and society	•Implementation of Salvinomics could have increased inequalities and economic instability. •Institutional reforms could have destabilized Salvini's electoral base.	•Qualitative study •Focused on potential outcomes rather than realized impacts.	•Assumes direct causality between political strategies and economic outcomes •Potential biases in interpreting political motivations and strategies.
2	Lesley H.	Life	To assess	•Voters	2021	Data	Relations	Public	SSM -	Peer-	Quantita	Analysis	•Examin	•Change	United	Resident	•Counties with	•Ecologic	Demograp

7	Curtis et.al. (38)	expectancy and voting patterns in the 2020 U.S. presidential election	the association between changes in life expectancy and voting patterns in the 2020 U.S. Presidential election.	•Demographic factors •Economic factors •Social factors		from 1980 to 2014 for life expectancy, 2020 for voting patterns.	hip between voting/political orientation/ behaviour and health indicators	Health, Political Science	Population Health	reviewed journal research article	tive research	of county-level life expectancy data and voting results.	ation of changes in life expectancy and their association with voting patterns. •Consideration of demographic, social, and economic variables.	in life expectancy •Proportion of votes •Demographic factors •Economic factors	States	s of 3110 U.S. counties	less positive changes in life expectancy were more likely to vote for the Republican candidate. •An increase in life expectancy was associated with an increase in the Republican vote share from 2016 to 2020.	al study: limits the ability to associate individual voting behaviors with health outcomes. •Limited by available data sources	hic factors, Economic factors
28	Denilson Bandeira Coêlho et.al. (39)	Mecanismos de difusão de políticas sociais no Brasil: uma análise do Programa Saúde da Família	Explore the diffusion of social health policies in Brazil, specifically the Family Health Program (PSF), by analyzing the roles of internal and external factors in the adoption of the program by local governments.	•Difusão de políticas •Programa Saúde da Família (PSF) •Governos municipais •Competição política •Análise de sobrevivência	2016	Data from 1997 to 2010	Relationship between voting/political orientation/ behaviour and health indicators	Public Health, Political Science	Revista de Sociologia e Política	Peer-reviewed journal research article	Quantitative analysis	Event History Analysis (EHA)	•Examination of the adoption rates of the PSF across municipalities •Analysis of political competition, ideological alignment, and demographic factors •Evaluation of horizontal (between municipalities)	•Political competition •Ideological alignment •fiscal capacity •Demographic factors •Adoption of the PSF by municipalities	Brazil	5,560 municipalities in Brazil	•Political competition and ideology significantly influence the adoption of the PSF. •Municipalities with left-wing mayors are more likely to adopt the PSF.	•Limited by the availability and quality of data on political and fiscal variables. •Focuses primarily on quantitative factors	Socio-economic variability among municipalities, Differences in local administrative capacity and healthcare infrastructure.

													and vertical (from federal to local governments) policy diffusion mechanisms.						
29	M. Shaw, D. Dorling, G. Davey Smith. (40)	Mortality and Political Climate: How Suicide Rates Have Risen during Periods of Conservative Government, 1901-2000	To investigate the relationship between political regimes and suicide rates, specifically examining how suicide rates have risen during periods of Conservative government in Australia and the United Kingdom.	•Suicide rates •Political climate •Conservative government •Public health •Epidemiology •Political regimes	2002	Data from 1901 to 2000	Relationship between voting/political orientation/ behaviour and mental health indicators	Public Health, Political Science Mental health	Journal of Epidemiology and Community Health	Peer-reviewed journal research article	Observational study	Comparative analysis of suicide rates	•Association between political regimes and suicide rates •Comparison of suicide rates during periods of Conservative and Labor government rule	•Suicide rates •Political regime (Conservative vs. Labor) •Economic condition s (GDP changes) •War periods •Availability of sedatives	Australia, United Kingdom	National populations of Australia and the United Kingdom	•Higher suicide rates were observed during periods of Conservative government in Australia •The effect was strongest when both levels of government were Conservative. •Similar patterns were observed in the United Kingdom	•The study does not explore underlying mechanisms in detail. •Limited by the availability and consistency of historical data.	Economic conditions War periods Changes in the role of women and their economic participation
30	Marta Castilho et.al. (41)	Negacionismo e o papel dos fatores políticos para a	To analyze the role of political orientatio	•Covid-19 •Political factors •Socioeconomic	2023	Data from Covid-19 pandemic in	Relationship between voting/political orientatio	Public Health, Political Science	Nova Economia	Peer-reviewed journal research	Econometric analysis	Econometric models	•Examination of the relationship between	•Political orientation •Socioeconomic factors	Brazil	Municipalities in Brazil	•Higher Covid-19 mortality rates in municipalities with higher support for		

		mortalidade por Covid-19 no Brasil	n, particularly support for President Bolsonaro, in the mortality rates of Covid-19 in Brazil.	inequalities •Mortality •Social distancing		2020 to October 202	n/ behaviour and vacination			article			political orientation and Covid-19 mortality rates. •Evaluates the "Bolsonaro effect" on Covid-19 mortality	•Covid-19 mortality rate			Bolsonaro. •Population mobility is a significant transmission channel for the disease. •Political denialism does not significantly affect the complete vaccination rate		
31	Anton Gollwitzer et.al. (42)	Partisan differences in physical distancing are linked to health outcomes during the COVID-19 pandemic	To investigate how political partisanship affects physical distancing behaviors during the COVID-19 pandemic and its subsequent impact on infection and fatality growth rates.	•COVID-19 •Physical distancing •Partisanship •Political identity •Public health •Infection rates •Mortality rates •Republican •Democrat	2020	Data from March to May 2020	Relationship between voting/political orientation/ behaviour and health indicators	Public Health, Political Science	Nature Human Behaviour	Peer-reviewed journal research article	Observational study	Multi-level mixed-effects models, mediation analyses, Geotracking data	•Examination of partisan differences in physical distancing. •Assessment of the impact of partisan differences on COVID-19 infection	•Physical distancing •Partisanship •COVID-19 infection and fatality growth rates	United States	Approximately 3,025 US counties	•Counties that voted for Trump exhibited 14% less physical distancing than those that voted for Clinton. •Partisanship was more strongly associated with physical distancing than other factors like population density, median income •Reduced physical distancing in pro-Trump counties was linked to higher infection and fatality growth rates.	•Limited to the United States •Did not account for the role of face masks or other preventative behaviors.	Population density, median income, state policies, etc.)
32	Haejoo Chung, Carles Muntaner. (43)	Politics, welfare regimes, and population health:	Determine the impact of political and welfare	•Welfare state •Politics of health care •Public	2006	1960-1994	Relationship between voting/political orientatio	Public Health, Political Science	Social Science & Medicine	Peer-reviewed journal research	Ecological study	Time-series multivariate regression model	Impact of political orientation and welfare state	•Infant mortality rate •Under-five mortality	Wealthy OECD countries (n=19)	Populations of 19 wealthy OECD countries	•Political and welfare variables associated with nfant and child health indicators	•Limited qualit of data •Don´t design the study	GDP per capita, Gini coefficient, social security

		Controversies and evidence	state variables on low birth weight rate, infant mortality rate, and under-five mortality rate in wealthy OECD countries	medical care •Infant mortality •Under-five mortality •Low birth weight •Comparative			n/ behaviour and health indicators			article			variables on child health indicators	rate •Low birth weight rate •Percentage of vote •Medical coverage			•Votes obtained by social democratic or labor parties related with low birth weight rate	for causal mechanisms	transfers
33	Subramanian Tsuyoshi et.al. (44)	Political Ideology and Health in Japan: A Disaggregated Analysis	Examine the association between political ideology and health status in Japan, investigating whether individuals with conservative political beliefs report better health and lower smoking rates compared to those with progressive beliefs.	•Political ideology •Health •Japan •Self-rated health •Smoking	2010	Data from 2000-2003, 2005, and 2006 Japan General Social Surveys	Relationship between voting/political orientation/ behaviour and health indicators	Public Health, Political Science	Journal of Epidemiology and Community Health	Peer-reviewed journal research article	Empirical Analysis using Survey Data	Logistic regression models	•Analysis of self-rated health and smoking status •Political ideology measured on a 5-point scale from 'left' to 'right'	•Political ideology •Self-rated poor health •Smoking status	Japan	Individuals from the Japan General Social Survey from 2000 to 2006	•Inverse association between political ideology and both self-rated poor health and smoking status. •Conservatives were less likely to report poor health (OR 0.86) and less likely to smoke (OR 0.80). •Political ideology might be a marker for several latent values and attitudes beneficial for health.	•Does not explore the underlying mechanisms by which political ideology influences health. •Causation is not fully addressed	Age, Sex, Education, Income, Occupational Status, Survey period

34	Paolo Nicola Barbieri, Beatrice Bonini. (45)	Political orientation and adherence to social distancing during the COVID-19 pandemic in Italy	To document how political orientation influences adherence to social distancing measures during the COVID-19 pandemic in Italy, using province-level geolocation data.	•COVID-19 •Coronavirus •Political belief •Protest vote •Geolocation	2021	Data from February to June 2020	Relationship between voting/political orientation/ behaviour and health indicators	Public Health, Political Science	Economia Politica	Peer-reviewed journal research article	Observational study	Econometric modeling	Examination of political orientations and their effect on social distancing behaviors.	•Social distancing compliance •Political orientation •Protest vote share. •COVID-19 cases and deaths.	Italy	Residents in Italian provinces	•Provinces with higher support for extreme right-wing parties showed lower compliance with social distancing measures. •Provinces with higher protest votes similarly showed lower compliance with social distancing •Higher compliance with social distancing was observed in provinces with more support for M5S •Political (mis)belief and discontent significantly influenced compliance with government lockdown measures.	•The study is limited to Italy	Socioeconomic and demographic characteristics
35	Viji Diane Kannan, Peter J. Veazie. (46)	Political orientation, political environment, and health behaviors in the United States	To examine the associations of political orientation and political environment with various health behaviors in the United	•Health behavior •Politics •Psychology •Exercise •Diet •food •Flu vaccine •Tobacco smoking •Alcohol drinking • Health	2018	Data from 2005 to 2012	Relationship between voting/political orientation/ behaviour and health indicators	Public Health, Political Science	Preventive Medicine	Peer-reviewed journal research article	Observational study	Logistic regression models.	•Examination of health behaviors such as health information search, flu vaccination, excessive alcohol consump	•Political orientation •Political environment •Health behaviors	United States	Adult sample from the ANHCS	•Democrats/ liberals had higher odds of cigarette smoking and excessive drinking compared to Republicans/conservatives. •Republicans/conservatives ate fewer servings and varieties of fruit and	•The study relies on self-reported data, which may be subject to reporting biases. •The findings may not generaliz	Socio-demographic covariates and state and county political environments.

			States.	information									tion, tobacco consumption, exercise, and dietary patterns. •Analysis of individual political orientation and political environment				vegetables, ate more high-fat and processed foods, and engaged in less in-depth health information searches compared to Democrats/liberals. •Conservatives had lower odds of exercise participation than liberals, and Republicans had lower odds of flu vaccination.	e beyond the United States due to the specific political and cultural context.	
36	D Hsiehchen, M Espinoza, P Slovic. (47)	Political partisanship and mobility restriction during the COVID-19 pandemic	Evaluate the relationship between compliance to non-pharmaceutical interventions and political party affiliations, specifically focusing on Republican identification	•Political orientation •Mobility restriction •COVID-19 pandemic •Non-pharmaceutical interventions •Republican affiliation	2020	March and April 2020	Relationship between voting/political orientation/behaviour and vacination	Public Health, Political Science	Public Health	Peer-reviewed journal research article	Quantitative assessment	Multivariable linear regression models to analyze the impact of Republican proportion and voter support for President Trump	Analyzed state-level data to assess the influence of political partisanship on adherence to NPIs	•Proportion of Republicans •Voter support for President Trump •Urban percentage •Socio-economic factors	United States	Individuals residing in different US states during the COVID-19 pandemic	•Negative correlation between Republican affiliation and NPI compliance •Highlight impact of political orientation on mobility restrictions	•Lack of broader theoretical relevance to political orientation and health	Potential biases introduced by using state-level data
37	José A. Tapia Granados.	Politics and health in eight	Analyze the influence	•Europe •Politics •Mortalit	2010	Period from 1950 to	Relationship between	Public Health, Political	Social Science &	Peer-reviewed	Comparative study of	Analysis of mortality	•Life expectancy at	•Political regime •Mortality	Eight European	General population of the	•Significant convergence in population	Complexity of the determin	Economic conditions Social

	(48)	European countries: A comparative study of mortality decline under social democracies and right-wing governments	of political regimes (social democracies vs. right-wing governments) on mortality decline in eight European countries.	y •Life expectancy •Welfare state •Southern Europe •Nordic countries		2000	voting/political orientation/ behaviour and health indicators	Science	Medicine	journal research article	mortality rates and life expectancy data	rates and life expectancy data from various sources	birth •Infant mortality rates •Correlations between years of social democracy in office and health indicators	rates •Life expectancy •Infant mortality rates	countries	eight European countries	health indicators among the eight countries despite different political regimes •Mean decadal gains in longevity were higher in Southern European countries •Political regime, health expenditure, and type of welfare state do not appear to be major determinants of mortality decline	ants of mortality trends	policies Historical and cultural differences among the countries
38	Vicente Navarro et.al. (49)	Politics and health outcomes	Examine the complex interactions between political traditions, policies, and public health outcomes, and to determine whether different political traditions have been associated with systematic patterns	•Political traditions •Health outcomes •Redistribution policies •Infant mortality •Life expectancy	2006	1950 to 2000	Relationship between voting/political orientation/ behaviour and health indicators	Public Health	The Lancet	Peer-reviewed journal research article	Empirical research study with an ecological study design.	Heuristic framework and bivariate Pearson correlation coefficients to analyze political, economic, social, and health variables	•Groups countries into four political traditions: social democratic, Christian democratic, liberal, and authoritarian conservative. •Analyzes how these political traditions and their associate	•Political variables (voter participation, voter partisanship, time in government by political parties) •Labour market data •Welfare state policies •Health outcomes	Wealthy OECD countries	Populations of 19 wealthy OECD countries	•Political ideologies of governing parties significantly affect health indicators. •Parties with egalitarian ideologies tend to implement redistributive policies, leading to better health outcomes •Redistributive policies are positively associated with health outcomes.	Limited by the availability of comparable data for the entire period	Variability in political ideologies and policies within the same political tradition over time

			in population health over time.										d policies impact health outcomes						
39	Songul Cinaroglu. (50)	Politics and Health Outcomes: A Path Analytic Approach	To determine the relationship between politics, labor and welfare state indicators, economic inequality, and health outcome indicators.	•Health outcomes<br>•Path analysis<br>•Politics	2019	Data from 2015	Relationship between voting/political orientation/behaviour and health indicators	Public Health, Political Science	International Journal of Health Planning and Management	Peer-reviewed journal research article	Path Analytic Approach	Path analytic model	•Highlights the strong relationship between voter partisanship, employment rate, satisfaction with social security and health services, and health outcomes<br>•Emphasizes the impact of socially inclusive policies	•Political variables (voter participation, voter partisanship, time in government by political parties)<br>•Satisfaction from social security<br>•Health outcomes	Turkey	Population of the 81 provinces of Turkey	•Significant relationship exists between voter partisanship and health outcomes.<br>•Increased voter support for the ruling party (AKP) is associated with higher health services	•Focuses solely on the post-reform period and does not consider the pre-reform period<br>•Examines the impact of political orientation in Turkey,	Complex interplay between various socio-economic factors and political orientation
40	Constantinos Alexiou, Emmanouil Trachanas. (51)	Politics, Government Health Expenditure, and Infant Mortality: Does Political Party Orientation Matter?	To explore the relationship between government political party orientation and infant mortality,	•Health expenditure<br>•Infant mortality<br>•Panel data<br>•Political party orientation	2021	Data from 2000 to 2018	Relationship between voting/political orientation/behaviour and health indicators	Public Health, Political Science	International Journal of Social Economics	Peer-reviewed journal research article	Control for heterogeneous parameters across countries and quantiles	Panel Quantile	•Impact of political party orientation (right, center, left) on infant mortality.<br>•Analyzes the mediatin	•Political party orientation<br>•Government health expenditure<br>•Income inequality<br>•Unemployment<br>•Education	15 countries from the G20 group.	Populations of the 15 G20 countries.	•Political party orientation significantly affects health outcomes<br>•Left-wing parties associated with better health outcomes.<br>•Government health expenditure plays a main	•Limited scope to G20 countries<br>•Does not explore pre-reform periods or other historical contexts.	•Socio-economic and political heterogeneity<br>•Potential unmeasured variables

			examining how different political orientations influence health outcomes through government health expenditure.										g effect of government health expenditure.	•Infant mortality.			role in reducing child mortality. •Redistributive policies contribute to better health outcomes.		
41	Carles Muntaner et.al . (52)	Politics, Welfare Regimes, and Population Health: Controversies and Evidence	Synthesize the evidence on how political traditions and welfare state characteristics influence population health	•Population health •Politics Political tradition Welfare state •Democracy •Globalisation	2011	Data from multiple studies	Relationship between voting/political orientation/ behaviour and health indicators	Public Health, Political Science	Sociology of Health & Illness	Peer-reviewed journal research article	Systematic Literature Review	Review of empirical and comparative studies using databases	•Examines the health impacts of different political traditions (left-wing, egalitarian, etc.)	•Political tradition •Welfare state characteristics •Democracy indicators •Health outcomes	OECD nations	Populations from multiple countries	•Left and egalitarian political traditions have the most positive effects on population health. •Advanced and liberal democracies are associated with better health outcomes. •Globalization is often negatively associated with population health.	•Limited focus on non-OECD countries. •Inconsistent results for relative health inequalities. •Need for more comprehensive and longitudinal studies.	•Potential unmeasured variables influencing health outcomes.
42	Everton Emanuel Campos de Lima et.al. (53)	Presidential election results in 2018-2022 and its association with excess mortality during the 2020-2021 COVID-	To evaluate the association between excess mortality and political partisanship in	•Excess Mortality •COVID-19 Pandemic •Politics •Presidential Election •Brazil	2024	Data from 2020 to 2021	Relationship between voting/political orientation/ behaviour and health indicator	Public Health, Epidemiology, Political Science	Cadernos de Saúde Pública	Peer-reviewed journal research article	Observational study	Spatial regression models and correlation analysis	•Analysis of the relationship between excess mortality and voting patterns	•Excess mortality •First-round votes for Bolsonaro (2018 and 2022) •Socioeconomic and health service	Brazil	Brazilian municipalities	•Positive association between the percentage of votes for Bolsonaro and excess mortality in Brazilian municipalities during the COVID-19 pandemic.	•Potential ecological fallacy due to the use of aggregated data. •Lack of updated demographic variables.	Consideration of various control variables including socioeconomic factors, health services availability

		19 pandemic in Brazilian municipalities	Brazil using municipal death certificates and first-round electoral results of Presidential elections in 2018 and 2022.				s							variables •Cultural variables			•The association suggests that Bolsonaro’s public stance and rhetoric against pandemic measures influenced the excess mortality rates.	•Limited consideration of other factors that may influence voter loyalty.	, and cultural variables.
43	Richard Kimball, Michael Wissner. (54)	Religion, Poverty, and Politics: Their Impact on Women’s Reproductive Health Outcomes	Explore the relationships between social determinants of health, including religion, voting patterns, child poverty, and income inequality, on women's reproductive health outcomes in the U.S.	•Community health nursing •Geographic Information systems (GIS) •Health disparities •Health policy •Population health outcomes •Reproductive health •Social determinants of health •Social justice •Socioeconomic factors •Women’s health	2015	Data from 2007-2008	Relationship between voting/political orientation/ behaviour and health indicators	Public Health, Political Science	Public Health Nursing	Peer-reviewed journal research article	Multiple regression analysis	Secondary data analysis using state-level data	Examines the impact of social determinants such as religion, voting patterns, child poverty, and income inequality on abortion rates, teen birth rates, and infant mortality rates	•Infant mortality rate (IMR) •Teen birth rate •Abortion rate •Religiosity •GINI coefficient •Child poverty rate •Voting patterns	United States	State-level populations across the 50 U.S. states	•Higher infant mortality rates are associated with higher religiosity scores. •Lower abortion rates are associated with voting conservatively •Higher teen birth rates are associated with higher child poverty rates and voting conservatively.	•Limited to U.S. data from 2007-2008 •Focuses on specific reproductive health outcomes without broader health indicators.	•Unmeasured variables influencing health outcomes

44	Johan P. Mackenbach, Martin McKee. (55)	Social-Democratic Government and Health Policy in Europe: A Quantitative Analysis	Examinates the effects of social-democratic government participation on indicators of preventive health policy and population health outcomes in Europe	•Social-democratic government •Health policy •Preventive health •Political orientation •Europe	2013	Data from 1946 to 2008	Relationship between voting/political orientation/ behaviour and health indicators	Public Health, Political Science	International Journal of Health Services	Peer-reviewed journal research article	Quantitative analysis	Regression models	•investigates the relationship between cumulative years of social-democratic government participation and various health policy performance indicators •Compares health policies and outcomes under social-democratic regimes with those under other political regimes.	•Type of government (social-democratic vs. others) •Health outcomes	Various European countries	General population of the European countries	•Social-democratic countries tend to have better health outcomes. •Health indicators like male smoking prevalence, alcohol consumption, and road safety measures were better in social-democratic countries.	•Complexity of establishing direct causal relationships •Variability in data collection methods	Differences in socio-economic factors, healthcare systems
45	Brittany N. Morey et.al. (56)	Symbolic disempowerment and Donald Trump's 2016 presidential election:	To examine the effect of the 2016 U.S. presidential election on the	•Mental health •U.S. presidential election •Race •Ethnicity •State	2021	Data from 2011 to 2018	Relationship between voting/political orientation/ behaviour and	Public Health, Political Science Mental health	Social Science & Medicine	Peer-reviewed journal research article	Quantitative analysis	Difference-in-differences analysis using negative binomial regressio	•Examination of trends in poor mental health days before and after	•Poor mental health days in the past 30 days •Race/ethnicity (non-	United States	Participants from BRFSS survey	•White populations in Clinton states reported more poor mental health days in response to the post-election period compared	•The study relies on self-reported data, which may be subject to	Socioeconomic variables, county-level demographic characteristics, health

		Mental health responses among Latinx and white populations	mental health of Latinx and white populations, considering race/ethnicity, language of interview, and state-level support for Trump or Clinton.	•Population health •National sociopolitical environment			mental health indicators					n models	the 2016 U.S. presidential election •Analysis of the impact of the election on six population categories based on race/ethnicity, language, and state political environment	Latinx white and Latinx populations) •Language of interview (English or Spanish) •State political environment (voted for Trump or Clinton)			to white populations in Trump states. •English-speaking Latinx people living in Trump states experienced higher than expected poor mental health •Spanish-speaking Latinx people, by contrast, reported fewer poor mental health	reporting biases. •The study design does not allow for causal inference. •Potential ecological fallacy due to aggregated data.	status and State political environment
46	Sarah Kohler Isabell Koinig. (57)	The Effect of Science-Related Populism on Vaccination Attitudes and Decisions	Examine the relationship between science-related populism and individuals' attitudes towards vaccination, presuming that science-related populism influences individual response	•Health communication •Vaccination hesitancy •Science-related populism •Vaccination confidence •Collective responsibility	2023	2021	Relationship between voting/political orientation/ behaviour and vacination	Public Health, Political Science	Journal of Behavioral Medicine	Peer-reviewed journal research article	Cross-sectional study using survey data	Binomial logistic regressions, linear regression models	Assesses how science-related populism influences vaccination attitudes, focusing on COVID-19 and MMR vaccinations.	•Vaccination attitudes •Science-related populism •Health consciousness •Demographic variables	Germany and Austria	Participants from Germany and Austria (n = 870)	•Science-related populism significantly influences attitudes towards COVID-19 and MMR vaccinations •Decreased vaccination confidence and collective responsibility •Increased complacency and perceived constraints.	•Limited to Germany and Austria	Unmeasured variables influencing vaccination attitudes

			s towards different vaccinations.															
47	Majdi M. Sabahelzain et.al. (58)	The Politics of Covid-19 Vaccine Confidence	Examine how political factors such as government handling of the pandemic, populism, and vaccine nationalism influence public confidence in COVID-19 vaccines.	•Vaccine confidence •COVID-19 •Political factors •Government response •Populism •Vaccine nationalism	2021	2021	Relationship between voting/political orientation/ behaviour and vacination	Public Health, Political Science	Current Opinion in Immunology	Peer-reviewed journal research article	Review and synthesis of existing studies	Qualitative analysis	Assesses how different political dynamics influence public attitudes towards COVID-19 vaccines.	•Public confidence in vaccines •Government response to COVID-19 •Populist rhetoric •Vaccine nationalism	Philippines, Brazil, the USA, and European nations	General populations in the mentioned countries	Public confidence in COVID-19 vaccines is significantly influenced by perceptions of government handling of the pandemic	•The study primarily focuses on COVID-19 vaccines, not others	Media influence and pre-existing health beliefs.
48	Tymor Hamamsy et.al.(59)	Viewing the US presidential electoral map through the lens of public health	To perform a comprehensive analysis of the relationship between voting patterns and over 150 different public health and wellbeing variables at the county	•Health •Disease •Mortality •Voting patterns •Public health •Wellbeing •Presidential election	2021	Data from 1980 to 2016	Relationship between voting/political orientation/ behaviour and health indicators	Public Health, Political Science	PLOS ONE	Peer-reviewed journal research article	Quantitative analysis	Pearson correlations, linear mixed models, multivariate linear models, lasso regression	•Investigates the relationship between voting patterns and a wide range of public health variables, including chronic diseases, mortality rates, healthcare costs, insurance	•Voting patterns •Chronic disease rates •Mortality rates •Healthcare costs •Insurance rates •Health behaviors •Socio-economic status •Demographic factors	United States	US counties	•Counties voting Republican in the 2016 election had worse overall health outcomes than those voting Democrat. •Highlights significant associations between various health measures and voting patterns	•Does not establish causality due to its observational design •Focuses on associations rather than direct causal links.	Socio-economic factors, demographic composition, healthcare access, health behaviors

			level in the US										rates, and health behaviors<br>•Focus on battleground states and counties that flipped from Democrat to Republican.						
49	Martín J, Correa M et.al. (60)	Democratic Quality and Excess Mortality During the COVID-19 Pandemic	Analyze how democratic quality influenced COVID-19 excess mortality in 80 countries before vaccine rollout. Evaluate different dimensions of democracy (electoral process, government functioning, political participation, political	COVID-19, Coronavirus, Excess mortality, Democracy index, Democratic quality, Efficient autocracies	2024	2024	Relationship between voting/political orientation/ behaviour and vacination	Public Health, Political Science	Scientific Reports (Nature)	Peer-reviewed journal research article	Cross-sectional statistical analysis	Multiple linear regression models analyzing excess mortality, democracy indices, and control variables (health spending, overweight population, population over 65, globalization index, income levels, temperat	•Evaluates democratic quality<br>•Uses excess mortality as key indicators<br>•Accounts for political culture	•Excess mortality per million<br>•Electoral process and pluralism<br>•Government function<br>•Political participation	80 countries on five continents	National populations of each of the 80 countries	•Higher democratic quality is associated with lower excess mortality during the pandemic.<br>•The political culture dimension had the strongest inverse correlation with excess mortality (-326.50, $p < 0.001$)<br>•Countries with lower democracy index values had higher excess mortality.<br>•Government function, electoral process, and	•Limited focus on electoral behavior and its direct influence on health policies.<br>•No in-depth exploration of specific health policy decisions<br>•Lack of data on public compliance with government measures in democrat	•Data manipulation in authoritarian regimes may lead to underreported COVID-19 deaths.<br>•Differences in healthcare infrastructure across countries may influence excess mortality beyond political factors.<br>•Cultural attitudes toward

			culture, and civil liberties).									ure).					civil liberties also showed significant inverse relationships with mortality rates.	ic vs. autocratic states.	health interventions could mediate the relationship between democracy and mortality.
50	Phifer, Benjamin (61)	How Political Orientation Shapes Sugar Consumption and Resistance to Sugar-reducing Nudges	Investigates whether political orientation influences sugar consumption.	Political Orientation;<br>Nudge;<br>Sugar Consumption;<br>Political Psychology	2024	Data collected from 2021-2023	Relationship between voting/political orientation/ behaviour and health indicators	Public Health, Political Science	Washington State University	Doctoral Dissertation	Empirical study	Five empirical studies testing the relationship between political orientation and sugar intake.<br>Three additional studies assessing how liberals and conservatives react to sugar-reducing nudges.	Uses survey data, experimental designs, and regression models to explore trends.	•Political orientation<br>Political ideology<br>•Sugar consumption levels<br>•Response to sugar-reducing nudges	United States	General U.S. consumers	•No significant difference in sugar consumption between conservatives and liberals.<br>•Political ideology does affect reactions to sugar-reducing nudges.<br>•Conservatives resist nudges that restrict personal choice (e.g., school bans, taxation).<br>•Liberals are more supportive of interventions that promote healthier diets.	•Finds no direct evidence that conservatives consume more sugar<br>•Does not analyze long-term behavioral changes<br>•Limited exploration of cultural and socioeconomic factors	•Other factors influencing sugar consumption (e.g., socioeconomic status, regional dietary norms).
51	Oberlander, Jonathan (62)	Polarization, Partisanship, and Health in the United States	Examine how polarization and partisanship shape health policy in the U.S	Polarization;<br>Partisanship;<br>Health Policy;	2024	Data collected from 2000-2023	Relationship between voting/political orientation/ behaviou	Public Health, Political Science	Journal of Health Politics	Peer-reviewed journal research article	Mixed-methods policy analysis	Evaluates how health outcomes differ across partisan lines in	•Partisan divisions in health policy decisions<br>•Impact of	•Political polarization<br>•Political ideology<br>behaviors	United States	General U.S. population	•Political polarization has reshaped U.S. health policy, creating two-tiered healthcare systems between Republican and	•No longitudinal analysis of partisan effects on healthcar	•Economic disparities between states may influence health outcomes independen

			and Investigate how political ideology influences public health behaviors and healthcare access	Public Health; COVID-19; Affordable Care Act; Political Identity			r and health indicators					different states. Examines political rhetoric, media framing, and legislative debates around healthcare.	partisanship on public health behaviors •Federal vs. state policy divergence in healthcare	•Federal vs. state policy divergence •Health policy outcomes •Public health behaviors •Trust in health institutions			Democratic states. •Democratic-led states enacted stricter public health mandates (masking, social distancing, vaccine requirements). •Republican-led states opposed federal mandates, emphasizing "personal freedom" over public health measures. •Higher vaccination rates among Democrats; lower rates in Republican-majority counties.	e over multiple decades •Limited examination of socioeconomic inequalities within partisan health outcomes. •No cross-national comparisons to assess whether U.S. health polarization is unique compared to other democracies.	tly of partisanship. •Media framing and misinformation contribute to polarization in health attitudes.
52	Jevuks Matheus et.al. (63)	Political Turnover and Public Health Provision in Brazilian Municipalities	Estimate the effects of political turnover on municipal health indicators, and test whether changes in party leadership at the	Regression Discontinuity; Close Elections; Political Turnover; Public Health	2024	Health indicators from 2009, 2011, 2013, and 2015	Relationship between voting/political orientation/ behaviour and health indicator	Public Health, Political Science	De Gruyter – Economics	Peer-reviewed journal research article	Quasi-experimental study	Regression Discontinuity Design (RDD) applied to close municipal elections (margin of ±5%).	•Impact of political turnover on health administration •Long-term health system impacts behavior	•Political turnover •Margin of victory •Budgetary variables •Number of SUS health workers	Brazil	Brazilian residents	•Political turnover improves administrative health indicators (e.g., workforce numbers, primary care coverage) but not structural health outcomes (e.g., mortality rates). •Municipalities	•Does not assess long-term policy effects beyond three years. •Lacks qualitative insights into decision-making	•Economic factors (e.g., municipal revenue, federal health funding) might independently affect health outcomes. •Variations

			municipal level influence public health policies and workforce composition.									Analysis of health indicators (SUS workforce, immunization, primary care coverage, etc.).	s<br>•Municipal health governance mechanisms	per capita<br>•Primary health care coverage rates<br>•Child mortality rates<br>•Vaccination coverage rates<br>•Basic health units created			with turnover had better-qualified health managers.<br>•Increased establishment of basic health units in municipalities that experienced political turnover.	processes within municipal governments.<br>•No examination of the ideological impact of different political parties on health policies.	in municipal governance structures may influence policy implementation.<br>•Local health crises (e.g., disease outbreaks) could skew short-term results.
53	Ross Arena, et al. (64)	The Relationship Between the Lifestyle Health Index and Voter Turnout During the 2020 United States Presidential Election in the Context of Regional Cultures	Examine how population health status correlates with voter turnout in the 2020 U.S. presidential election. It also introduce and validate the Lifestyle Health Index (LHI) as a predictor of civic engagem	Unhealthy Lifestyle Behaviors;<br>Chronic Disease;<br>Social Vulnerability<br>Public Health	2024	Health data from 2021	Relationship between voting/political orientation/ behaviour and health indicators	Public Health, Political Science	Public Health in Practice (Elsevier)	Peer-reviewed journal research article	Descriptive, cross-sectional, retrospective analysis.	County-level health and voting data analysis from multiple public databases, with statistical correlations between LHI scores and 2020 voter turnout. Also use of the American Nations regional	•Relationship Between Health and Civic Engagement<br>•Political and Geographic Trends in Health and Voter Turnout<br>•Role of Health Professionals in Civic Engagement	•Lifestyle Health Index<br>•County-level demographics.<br>•Regional cultural identity<br>•Voter turnout percentage<br>•Comparison of voter turnout between Democratic and Republican countie	United States	All U.S. counties (n=3,062)	•Higher rates of chronic disease and unhealthy behaviors were strongly linked to lower voter turnout.<br>•Republican-majority counties had higher LHI scores and lower voter turnout.<br>•Democratic-majority counties had lower LHI scores and higher voter turnout.<br>•The relationship between health	•Does not establish causality between health status and voter turnout.<br>•Limited control for social and economic policy influences on health outcomes.<br>•No analysis of voter suppression or	•Income and education levels, which are known to influence both health and voter turnout.<br>•Access to healthcare services, which could mediate the effects of poor health on voting behavior.<br>•Geographic differences

			ent.									culture model to Geospatial mapping of voter turnout and health disparities					and civic participation was consistent across all U.S. regional cultures.	policy barriers to electoral participation.	in voting laws and accessibility.
